# Supplementary material for: Adaptation is maintained by the parliament of genes
Source: Nat Commun. 2019 Nov 14;10:5163. doi: 10.1038/s41467-019-13169-3 (PMC6856117; doi:10.1038/s41467-019-13169-3)
Supplement: Supplementary file 1 — Supplementary Information [file 41467_2019_13169_MOESM1_ESM.pdf]

Supplementary Information

Adaptation is maintained by the parliament of genes

Scott, T.W., & West, S.A.

Supplementary Notes:

(1) Functional Forms Assumed in Figure 2b (Main Text) p1

(2) Equilibrium Models (Additional Figures) p2

(3) Sex Ratio Distortion p4

(4) Genomic Imprinting and Altruism p28

(5) Horizontal Gene Transfer and Public Goods p40

(6) Suppressor Conditionality p48

(7) Dynamics Models (Additional Figures) p51

(8) Real-World Estimates of Proportional Cabal Size p58

Supplementary Discussion:

(1) Relation to Gardner & Úbeda (2017) and Grafen's Formal Darwinism p60
Individual fitness maximisation: emergence versus maintenance

(2) Simple Selfish Genetic Elements vs Trait Distorters p63
Relation to Eshel (1984) and Eshel (1985)

(3) Relation to Cosmides & Tooby (1981): Coreplicons, Cabal & Commonwealth p68

Supplementary References p74

Supplementary Note 1

Functional Forms Assumed in Figure 2b (Main Text)

To generate Figure 2b (Main Text), we assumed the functional forms c_{trait}=0.8$k^{1.3}$ and t=T1k, where 0≤T1≤1. The parameter T1 mediates the rate at which the marginal transmission advantage dissipates, relative to the marginal individual cost of trait distortion, as the trait becomes increasingly distorted (k). This parameter (T1) is plotted along the *x* axis of Figure 2b (Main Text). We analytically derived the target trait distortion (k_{target}), for different values of c_{sup} and T1, by substituting our specific functional forms into the condition that specifies k_{target}: $\frac{dt}{dk}(1 - c_{trait}) = \frac{dc_{trait}}{dk}$. This gave: $\frac{d(T1\ k)}{dk}(1 - 0.8k^{1.3}) = \frac{d(0.8k^{1.3})}{dk}$, which simplifies to T1(1-0.8$k^{1.3}$)=1.04$k^{0.3}$, which was solved for k to give $k = k_{target}$. We derived the equilibrium trait distortion (k^*) by substituting each value of k_{target} into the condition for suppressor spread: c_{sup}<c_{trait}(k_{target}). Satisfaction of this condition implies that k^*=0; else, k^*=k_{target}.

Supplementary Note 2

Equilibrium Models (Additional Figures)

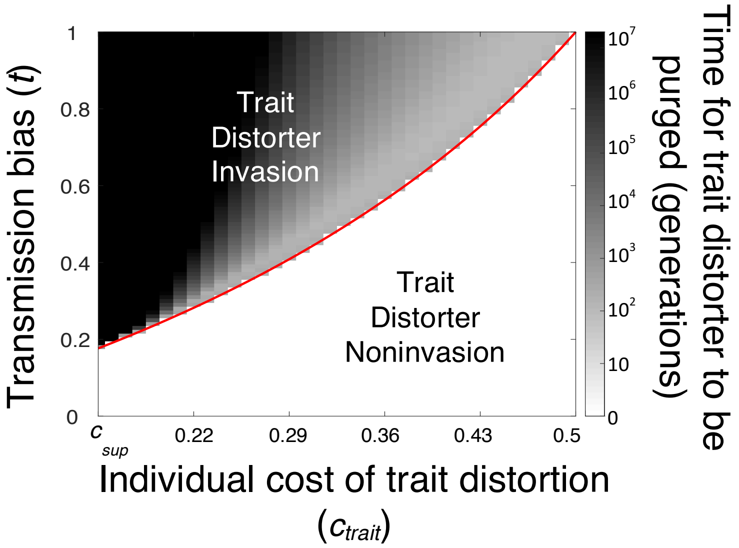

Supplementary Figure 1. Non-equilibrium trait distortion. A trait distorter (D_1) is introduced from rarity alongside its suppressor (S_1) (initial genotype frequencies: x_{00}=0.97, {x_{01},x_{10},x_{11}}=0.01). The trait distorter (D_1) is associated with some individual cost when expressed (c_{trait}) which is varied along the x axis (the cost of suppression is fixed at c_{sup}=0.15). The trait distorter (D_1) is also associated with a transmission bias at meiosis (t) which is varied along the y axis. We consider trait distorters that induce suppressor spread ($c_{sup}<c_{trait}$) and ask whether such trait distorters can cause appreciable trait distortion before they are ultimately suppressed and purged from the population. The red line plots the formula $t=c_{trait}/(1-c_{trait})$; above this line, trait distorters can spread from rarity. We plot the number of generations (on a $\log_{10}$ scale) until equilibrium is reached (trials that did not equilibrate by 20,000,000 generations were capped). We see that less costly trait distorters (c_{trait} only slightly greater than c_{sup}) can invade even with a relatively low transmission bias (t), and are purged at a very slow rate, causing extended non-equilibrium trait distortion. More costly trait distorters (c_{trait} large compared to c_{sup}) require a high transmission bias (t) to invade, and if they can invade, they are purged relatively quickly, causing shorter non-equilibrium trait distortion. Therefore, non-equilibrium trait distortion is either not-so-costly and extended, or costly and ephemeral, and so has limited impact on individual fitness maximisation in either case.

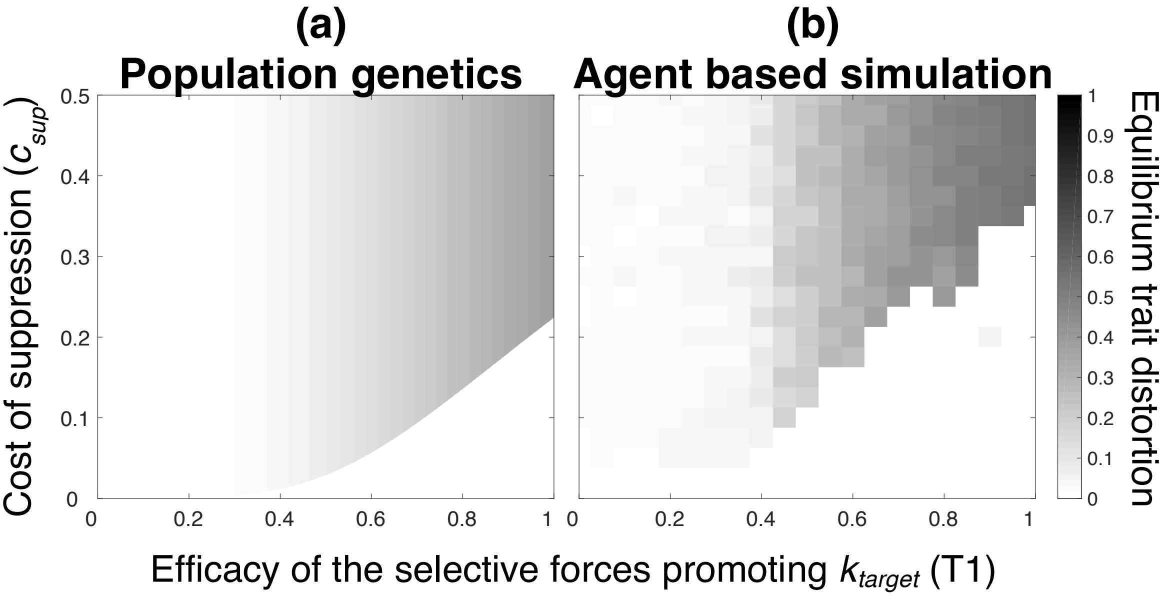

Supplementary Figure 2. Comparison of population genetic and agent based simulation results of the illustrative model. Trait distorter strength evolves in the presence of a suppressor of distortion (S_1) and the resulting equilibrium trait distortion ($0 \leq k^* \leq 1$) is plotted, using the following functional forms for trait distorter cost (c_{trait}) and transmission advantage (t): $c_{trait}(k) = 0.8k^{1.3}$ and $t(k) = T1k$, where $0 \leq T1 \leq 1$, and where T1 and c_{sup} are varied. T1 mediates the rate at which the marginal transmission advantage $\left(\frac{dt}{dk}\right)$ dissipates, relative to the marginal individual cost $\left(\frac{dc_{trait}}{dk}\right)$ of trait distortion, as the trait becomes increasingly distorted (k). Part (a) plots the equilibrium trait distortion under weak selection, as analytically derived from the population genetic model in the main text. Part (b) plots the equilibrium trait distortion as obtained by the agent based simulation detailed in *Methods: Agent-based simulation (single trait distorter locus)*, which does not assume weak selection, and which allows continuous variation at the trait and suppressor loci. There is good correspondence between the models; however, the region in which trait distorters are suppressed ($k^*=0$; bottom right) is larger in the agent based simulation, and outside of this region, the equilibrium trait distortion (k^*) is slightly greater in the agent based simulation. Discrepancy arises from the different assumptions about the strength of selection.

Supplementary Note 3

Sex Ratio Distortion

We examine sex ratio evolution in a diploid species, in a large outbreeding (panmictic) population, with non-overlapping generations, and where males and females are equally costly to produce. Fisher[1] and many others have shown that, in this scenario, individuals would be selected to invest equally in male and female offspring[2,3]. We assume genetic sex determination, with males as XY and females as XX[4].

We consider a selfish genetic element residing on an X chromosome, that may gain a propagation advantage by distorting the offspring sex ratio towards a greater production of females. The genes that do not gain a propagation advantage from female sex ratio bias reside on both the autosomes and the Y chromosome[5]. We focus on suppressors in the autosomes, for simplicity, and because this is the larger group of genes, constituting the majority within the parliament of genes[6]. Consequently, we focus our analyses on when an X driver and an autosomal suppressor can spread.

Our overall aim is to assess, given the potential for suppression, the extent that an X chromosome driver can distort the sex ratio away from the individual optimum. The individual optimum is taken to be the evolutionarily stable strategy (ESS) adopted by individuals in the absence of selfish trait distortion, which is an equal investment in offspring of both sexes.

We build our model in a step-wise manner, as described in the “Equilibrium Models” section of the main text. Aspects of questions 1-3 have been analysed before with respect to sex ratio, but we go over them here for the specific case of our model, and to elucidate the underlying selective forces. There is available data on the fitness consequences of sex ratio distortion and suppression, and so, in this case, we aim for a biologically realistic model that can be parameterised.

(1) Spread of a Trait distorter

We considered the spread, in the absence of suppression, of a selfish sex ratio distorter that skews offspring sex ratio towards females. In the literature, selfish X drivers are often denoted by *SR* (for *sex-ratio* distortion), with non-distorting rival alleles denoted by *ST* (for *standard*)[7]. However, we denote the trait distorting and non-distorting alleles respectively by D_1 and D_0 for consistency across our models. We assume that normal (D_0/Y) males produce X and Y sperm equally. The trait distorter (D_1) causes D_1/Y males to kill Y-bearing sperm, leading to a female-biased sex ratio[8-13]. In males with an unsuppressed trait distorter, its proportion of X-bearing sperm, and correspondingly, the proportion of its offspring that are female, is given by $0.5(1+k)$, where k denotes the proportion of Y-bearing sperm that are killed ($0<k\leq1$).

We assume that males with an unsuppressed trait distorter (D_1) suffer a fertility cost as a result of sperm death, and have a reduced ejaculate size of $1-k/2$, relative to 1 in all other males[14-19]. We assume that, each generation, each female copulates with λ random males, and that each sperm cell is equally competitive in the female’s

internal store. The likelihood of a male's sperm fertilising an egg (*fertility, F*) is given by his ejaculate size relative to the total amount of ejaculate that female has received. Letting l be the proportion of males in the present generation with an unsuppressed trait distorter, the fertility of those males with an unsuppressed trait distorter (F_{drive}), and the fertility of those without (F_{normal}), is given by:

$$F_{drive} = \sum_{i=1}^{\lambda} \frac{\left(1-\frac{k}{2}\right)}{\left(1-\frac{k}{2}\right)i+(\lambda-i)} l^{i-1}(1-l)^{\lambda-i}, \tag{1}$$

$$F_{normal} = \sum_{i=1}^{\lambda} \frac{1}{\left(1-\frac{k}{2}\right)(i-1)+(\lambda-i+1)} l^{i-1}(1-l)^{\lambda-i}. \tag{2}$$

There is no sperm competition, and therefore no fertility cost of sex ratio distortion, when females are singly mated ($F_{normal}=F_{drive}$ when $\lambda=1$). There is increased sperm competition at higher female mating rates, meaning the relative fertility cost of sex ratio distortion (F_{normal}/F_{drive}) increases and plateaus for high λ at $F_{normal}/F_{drive} = 1/(1-k/2)$.

The trait distorter has no fitness consequences for females, and so the condition for the spread of the trait distorter (D_1) allele is that D_1/Y males sire more female offspring than D_0/Y males. In the absence of suppression, D_1/Y males have $F_{drive}(1+k)/2$ female offspring, and D_0/Y males have $F_{normal}/2$ female offspring, meaning the trait distorter (D_1) is selected when:

$$F_{normal} / F_{drive} < (1+k). \tag{3}$$

The left-hand side of Supplementary Equation 3 gives the between-individual relative fertility cost of trait distortion and the right-hand side gives the within-individual relative transmission advantage of trait distortion. When we substitute our explicit fertility functions (Supplementary Equations 1 & 2) into Supplementary Equation 3, we find that Supplementary Equation 3 is always satisfied. Consequently, analogous to previous arguments, the distorting D_1 chromosome will always spread to fixation, irrespective of female mating frequency (λ)[20,21]. This distorts the offspring sex ratio, defined as the proportion of females, to $(1+k)/2$.

Previous models have relaxed some of our simplifying assumptions, allowing a fixed (mating rate (λ)-independent) cost of distortion, and allowing the female mating rate (λ) to change as the trait distorter spreads[5,8,14,17,22-27]. We have explored these factors and found that our general conclusions: (i) are not altered; and (ii) do not depend on the trait distorter (D_1) allele spreading all the way to fixation (Scott, unpublished).

(2) Spread of an autosomal suppressor

We assume that the sex ratio distorter can be suppressed by an autosomal allele (suppressor), as has been found in many *Drosophila* species[28-31]. We base our model upon the biology of *Nmy*, which suppresses the X chromosome trait distorter *Dox* in *Drosophila simulans*. *Nmy* works by RNAi-mediated destruction of the trait distorter's mRNA transcripts. *Nmy* is dominant, and only expressed in the presence of the trait distorter (*Dox*)[32-34].

For consistency across models, we denote the autosomal suppressor allele as S_1, and the wild type non-suppressor allele as S_0. We assume that the suppressor (S_1) is dominant, meaning individuals bearing at least one suppressor (S_1) allele suffer no sperm death and consequentially no fertility loss or sex ratio distortion. We assume that the suppressor (S_1) is only expressed in the presence of an active trait distorter (in D_1/Y males). When the suppressor is expressed it leads to a cost, which reduces the probability (V) that an individual survives from zygote to adult[35-38], from V_{normal}=1 in individuals without an active suppressor, to $V_{suppression}$=1-c_{sup} in individuals with one. The cost of suppression is a fixed cost (c_{sup}) of activating an RNAi pathway. Assuming alternatively that the suppression cost affects fertility rather than viability does not qualitatively change our results (Scott, unpublished).

We ask when an autosomal suppressor (S_1) will spread from rarity, given that an X chromosome trait distorter (D_1) is at fixation. Given that the suppressor only has phenotypic effects in D_1/Y males, it will spread from rarity if D_1/Y males bearing a suppressor (S_1) have more mated offspring than D_1/Y males lacking a suppressor (S_0/S_0). Assuming that the trait distorter and non-suppressor alleles are at fixation, and random mating, D_1/Y males with a suppressor will have $V_{suppression}*F_{normal}*(½)*((1+k)/2)$ mated female offspring, and $V_{suppression}*F_{normal}*(½)*((1-k)/2)$ mated male offspring, leading to a total of $V_{suppression}*F_{normal}*(¼)$ mated offspring. D_1/Y males lacking a suppressor will have a total of $2*V_{normal}*F_{drive}*((1-k)/2)*((1+k)/2)$ mated offspring. Suppressed D_1/Y males will therefore have more offspring, and the suppressor allele (S_1) will spread from rarity, when the following condition is satisfied:

$(F_{normal}/F_{drive})*(1/(1-k^2)) > (V_{normal}/V_{suppression})$. (4)

The overall cost of letting the trait distorter (D_1) go unsuppressed is a product of the costs to fertility (F_{normal}/F_{drive}) and offspring mating success ($1/(1-k^2)$). For a suppressor to spread, this must be greater than the viability cost of suppression ($V_{normal}/V_{suppression}$). Consequently, analogous to previous results, the suppressor (S_1) will only spread when the trait distorter (D_1) leads to appreciable trait distortion[36,39-42].

A previous model asked whether female-biased sex ratio distortion can select for compensatory evolution on autosomes, such that the autosomes evolve to encode a male-biased sex ratio in the absence of the trait distorter[43]. It found that compensatory evolution does not evolve when the female-biased sex ratio distorter is transmitted into female offspring with 100% certainty, as is the case for X drivers acting in males. This is why we did not allow compensatory strategies to evolve on autosomes in our model, and only allowed autosomes to suppress the trait distorter.

(3) Consequences for organism trait values

We turn to the question of how trait distorter-suppressor dynamics affect sex ratio. When both the trait distorter (D_1) and suppressor (S_1) are in a population, the genotypes they are in matters (epistasis), and so we explicitly track the frequencies of all 15 possible genotypes, with 15 recursions. The 15 equations represent the generational changes in each of the 15 possible genotypes. We let p_{fi} and q_{mi} be the proportion of the ith female genotype and the ith male genotype, respectively, in the

current generation (Supplementary Table 1). We let p_{fi}' and q_{mi}' be the frequencies of female and male genotypes in the next generation. The population sex ratio is given by the population proportion of females, $\sum p_f$. The equations are listed in Supplementary Table 2. We note that, in the absence of the trait distorter (D_1), population sex ratio evolves to 0.5, and after this, genotype frequencies remain constant over time (Hardy-Weinberg equilibrium).

Supplementary Table 1: Selection coefficients, drive values, and genotype frequency notation. For each male and female genotype, its proportion in the population at generation *t*, and its probability of maturing from a zygote to an adult (viability, *V*) is given. For each male genotype, the proportion of X chromosomes in its sperm store (drive), and its probability of successfully fertilising the female's egg cell after copulation (fertility, *F*), is given. Male fertility (*F*) depends on the number of mates each female has per generation (λ), and is written in full in Supplementary Equations 1 & 2. k gives the proportion of a male's Y bearing sperm that are killed, and c_{sup} gives the viability cost of trait distorter suppression.

		Females			Males	
		D_0/D_0	D_0/D_1	D_1/D_1	D_0/Y	D_1/Y
S_1 / S_1	***Proportion***	p_{f1}	p_{f4}	p_{f7}	p_{m1}	p_{m4}
	Fertility, F	/	/	/	F_{normal}	F_{normal}
	Viability, V	1	1	1	1	$1-c_{sup}$
	Drive	/	/	/	0.5	0.5
S_1 / S_0	***Proportion***	p_{f2}	p_{f5}	p_{f8}	p_{m2}	p_{m5}
	Fertility, F	/	/	/	F_{normal}	F_{normal}
	Viability, V	1	1	1	1	$1-c_{sup}$
	Drive	/	/	/	0.5	0.5
S_0 / S_0	***Proportion***	p_{f3}	p_{f6}	p_{f9}	p_{m3}	p_{m6}
	Fertility, F	/	/	/	F_{normal}	F_{drive}
	Viability, V	1	1	1	1	1
	Drive	/	/	/	0.5	$(1+k)/2$

Supplementary Table 2: Recursions detailing the change in proportion of each genotype across one generation (D_0 and D_1 segregating at trait locus). Notation is defined in Supplementary Table 1. T is the sum of the right sides of the system of equations such that ∑p=1. It normalises the recursions to ensure that gene frequency changes reflect proportions.

T p_{f1}' =	$(p_{f1} + 0.5\ p_{f2} + 0.5\ p_{f4} + 0.25\ p_{f5})\ (0.5\ p_{m1} + 0.25\ p_{m2})\ F_{normal}$
T p_{f2}' =	$((0.5\ p_{f2} + p_{f3} + 0.25\ p_{f5} + 0.5\ p_{f6})\ (0.5\ p_{m1} + 0.25p_{m2}) + (p_{f1} + 0.5\ p_{f2} + 0.5\ p_{f4} + 0.25\ p_{f5})\ (0.25\ p_{m2} + 0.5\ p_{m3}))\ F_{normal}$
T p_{f3}' =	$(0.5\ p_{f2} + p_{f3} + 0.25\ p_{f5} + 0.5\ p_{f6})\ (0.25p_{m2} + 0.5\ p_{m3})\ F_{normal}$
T p_{f4}' =	$(0.5\ p_{f4} + 0.25\ p_{f5} + p_{f7} + 0.5\ p_{f8})\ (0.5\ p_{m1} + 0.25\ p_{m2}) + (p_{f1} + 0.5\ p_{f2} + 0.5\ p_{f4} + 0.25\ p_{f5})\ (p_{m4}/2 + p_{m5}/4)\ F_{normal}$
T p_{f5}' =	$((0.25\ p_{f5} + 0.5\ p_{f6} + 0.5\ p_{f8} + p_{f9})\ (0.5\ p_{m1} + 0.25\ p_{m2}) + (0.5\ p_{f4} + 0.25\ p_{f5} + p_{f7} + 0.5\ p_{f8})\ (0.25\ p_{m2} + 0.5\ p_{m3}))\ F_{normal} + (p_{f1} + 0.5\ p_{f2} + 0.5\ p_{f4} + 0.25\ p_{f5})\ (1/2\ (1 + k)\ F_{drive}\ p_{m6} + 1/4\ p_{m5}\ F_{normal}) + (0.5\ p_{f2} + p_{f3} + 0.25\ p_{f5} + 0.5\ p_{f6})\ (p_{m4}/2 + p_{m5}/4)\ F_{normal}$
T p_{f6}' =	$(0.25\ p_{f5} + 0.5\ p_{f6} + 0.5\ p_{f8} + p_{f9})\ (0.25\ p_{m2} + 0.5\ p_{m3})\ F_{normal} + (0.5\ p_{f2} + p_{f3} + 0.25\ p_{f5} + 0.5\ p_{f6})\ (1/2\ (1 + k)\ F_{drive}\ p_{m6} + 1/4\ p_{m5}\ F_{normal})$
T p_{f7}' =	$(0.5\ p_{f4} + 0.25\ p_{f5} + p_{f7} + 0.5\ p_{f8})\ (p_{m4}/2 + p_{m5}/4)\ F_{normal}$
T p_{f8}' =	$((0.5\ p_{f4} + 0.25\ p_{f5} + p_{f7} + 0.5\ p_{f8})\ (1/2\ (1 + k)\ F_{drive}\ p_{m6} + 1/4\ p_{m5}\ F_{normal}) + (0.25\ p_{f5} + 0.5\ p_{f6} + 0.5\ p_{f8} + p_{f9})\ (p_{m4}/2 + p_{m5}/4)\ F_{normal})$
T p_{f9}' =	$(0.25\ p_{f5} + 0.5\ p_{f6} + 0.5\ p_{f8} + p_{f9})\ (1/2\ (1 + k)\ F_{drive}\ p_{m6} + 1/4\ p_{m5}F_{normal})$
T p_{m1}' =	$(p_{f1} + 0.5\ p_{f2} + 0.5\ p_{f4} + 0.25\ p_{f5})\ ((0.5\ p_{m1} + 0.25\ p_{m2})\ F_{normal} + 1/2\ p_{m4}\ F_{normal} + 1/4\ p_{m5}\ F_{normal})$
T p_{m2}' =	$(p_{f1} + 0.5\ p_{f2} + 0.5\ p_{f4} + 0.25\ p_{f5})\ ((0.25\ p_{m2} + 0.5\ p_{m3})F_{normal} + 1/2\ (1 - k)\ F_{drive}\ p_{m6} + 1/4\ p_{m5}\ F_{normal}) + (0.5\ p_{f2} + p_{f3} + 0.25\ p_{f5} + 0.5\ p_{f6})\ ((0.5\ p_{m1} + 0.25\ p_{m2})F_{normal} + 1/2\ p_{m4}\ F_{normal} + 1/4\ p_{m5}\ F_{normal})$
T p_{m3}' =	$(0.5\ p_{f2} + p_{f3} + 0.25\ p_{f5} + 0.5\ p_{f6})\ ((0.25\ p_{m2} + 0.5\ p_{m3})F_{normal} + 1/2\ (1 - k)\ F_{drive}\ p_{m6} + 1/4\ p_{m5}\ F_{normal})$
T p_{m4}' =	$V_{suppression}\ (0.5\ p_{f4} + 0.25\ p_{f5} + p_{f7} + 0.5\ p_{f8})\ ((0.5\ p_{m1} + 0.25\ p_{m2})F_{normal} + 1/2\ p_{m4}\ F_{normal} + 1/4\ p_{m5}\ F_{normal})$
T p_{m5}' =	$V_{suppression}\ ((0.5\ p_{f4} + 0.25\ p_{f5} + p_{f7} + 0.5\ p_{f8})\ (0.25F_{normal}\ p_{m2} + 0.5F_{normal}\ p_{m3} + 1/2\ (1\text{-}\ k)\ F_{drive}\ p_{m6} + 1/4\ p_{m5}\ F_{normal}) + (0.25\ p_{f5} + 0.5\ p_{f6} + 0.5\ p_{f8} + p_{f9})\ (0.5\ p_{m1} + 0.25\ p_{m2} + 1/2\ p_{m4}\ F_{normal} + 1/4\ p_{m5}\ F_{normal}))$
T p_{m6}' =	$(0.25\ p_{f5} + 0.5\ p_{f6} + 0.5\ p_{f8} + p_{f9})\ ((0.25\ p_{m2} + 0.5\ p_{m3})F_{normal} + 1/2\ (1 - k)\ F_{drive}\ p_{m6} + 1/4\ p_{m5}\ F_{normal})$.

To illustrate the logic of the equations, we derive one recursion explicitly. We derive the recursion for p_{m5}', which gives the frequency, in the next generation, of males bearing the trait distorter (D_1/Y) and one suppressor allele (S_1/S_0). We denote the

current generation as G1 and the next generation as G2. A mating between D_1/Y, S_0/S_0 males (at frequency p_{m6}) and D_1/D_1, S_0/S_1 females (at frequency p_{f8}) can give rise to individuals in G2 with our focal genotype D_1/Y, S_1/S_0. Of all matings between males and females in G1 ($\sum_{i=1}^{6}\sum_{j=1}^{9} p_{mi} * p_{fj}$), these matings occur in the proportion $(p_{m6} * p_{f8})/(\sum_{i=1}^{6}\sum_{j=1}^{9} p_{mi} * p_{fj})$ of cases. Copulation success, in which the egg is successfully fertilised to form a zygote, depends on the fertility of the male, which in our case is F_{drive} (Supplementary Equation 1). Of all the zygotes produced by the population in G1, our parents will contribute the proportion $(p_{m6} * p_{f8})/$ $(\sum_{i=1}^{6}\sum_{j=1}^{9} p_{mi} * p_{fj}))$ * (F_{drive} / $\sum_{i=1}^{6} p_{mi} F_{mi}$) of them, where $\sum_{i=1}^{6} p_{mi} F_{mi}$ gives average male fertility. These zygotes will have the focal offspring genotype (D_1/Y, S_1/S_0) if they inherit a YS_0 gamete from the father (with probability (1-k)/2) and a D_1S_1 gamete from the mother (with probability 1/2), meaning the proportion of zygotes in the population with the focal genotype, (D_1/Y, S_1/S_0), stemming from copulations between our focal parents, is $(p_{m6} * p_{f8})/(\sum_{i=1}^{6}\sum_{j=1}^{9} p_{mi} * p_{fj}))$ * (F_{drive} / $\sum_{i=1}^{6} p_{mi} F_{mi}$) * (1+$k$)/4. Finally, only the proportion $V_{suppression}$ of these focal zygotes (D_1/Y, S_1/S_0) will successfully mature to adulthood in G2, meaning the proportion of mature adults in G2 that have the focal genotype (D_1/Y, S_1/S_0) and arose from copulations between D_1/Y, S_0/S_0 males and D_1/D_1, S_0/S_1 females in G1 is given by $(p_{m6} * p_{f8})/(\sum_{i=1}^{6}\sum_{j=1}^{9} p_{mi} * p_{fj}))$ * (F_{drive} / $\sum_{i=1}^{6} p_{mi} F_{mi}$) * (1+$k$)/4 * ($V_{suppression}$ / $\sum p^* V$), where $\sum p^* V$ is the average viability of individuals. We simplify the expression by making the substitution $T = (\sum_{i=1}^{6}\sum_{j=1}^{9} p_{mi} * p_{fj})(\sum_{i=1}^{6} p_{mi} F_{mi})(\sum p * V)$, our normalisation factor. We now need to sum over all possible parental copulations that can give rise to D_1/Y, S_0/S_1 offspring. Doing so gives the frequency of the D_1/Y, S_0/S_1 genotype in the next generation, p_{m5}', written in full in Supplementary Table 2.

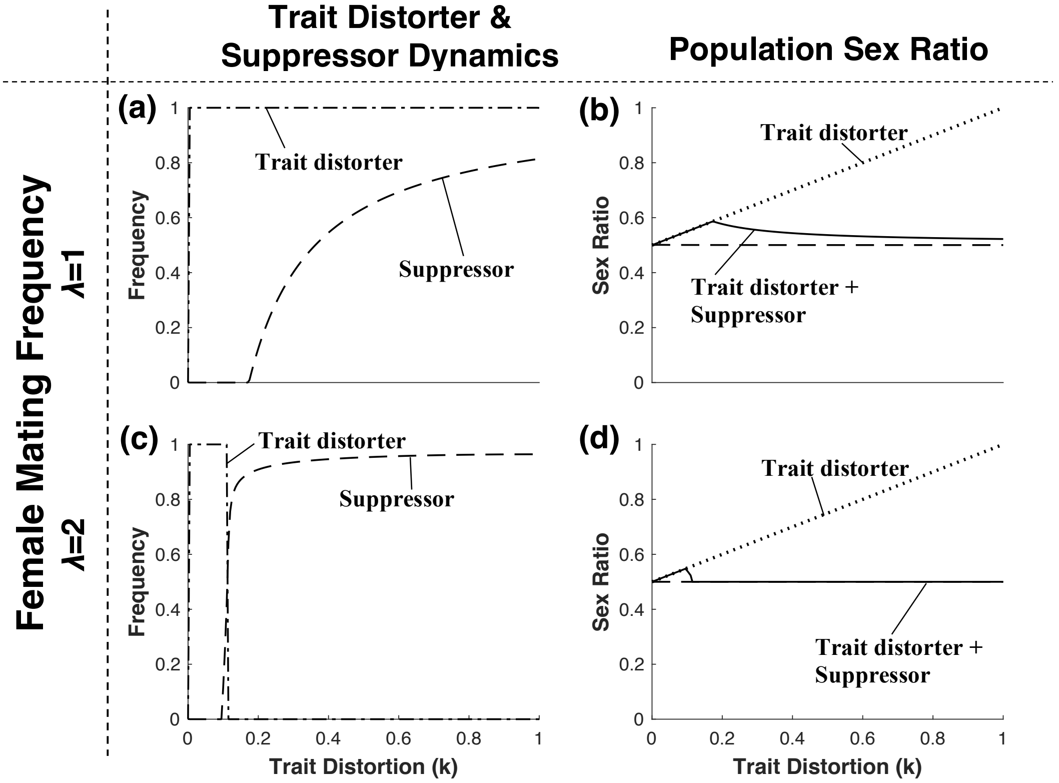

Supplementary Figure 3. Drive-suppressor coevolution and resulting sex ratio. A sex ratio distorter (D_1) and its suppressor (S_1) are introduced from rarity. The left-hand column shows the resulting equilibrium trait distorter (D_1; *dot-dash* line) and suppressor (S_1; dashed line) frequencies, for different trait distorter strengths ($0<k\leq1$). The right-hand column shows the resulting population average sex ratio. The dotted and dashed lines are plotted for reference, to show, respectively, the sex ratio ($(1+k)/2$) that arises in the absence of suppression, and the sex ratio (½) that arises in the absence of the trait distorter (D_1). The top row shows results for when females are singly mated (λ=1) and the bottom row shows results for when females are doubly mated (λ=2). The numerical solutions assume that the cost of suppression is c_{sup}=0.03. We see that equilibrium suppressor (S_1) frequency is greater for strong (higher-k) drivers, resulting in full (λ=2) or partial (λ=1) restoration of the individual optimal sex ratio (0.5) for strong (higher-k) drivers.

We iterated these recursions to find the trait distorter (D_1) and suppressor (S_1) frequencies, and the population sex ratio ($\sum p_f$), at equilibrium (Supplementary Figure 3). When we introduced both the trait distorter and suppressor at low frequencies,

we confirmed our above results that the trait distorter (D_1) initially spreads to fixation, and that the suppressor allele (S_1) only invades and reaches high frequencies if it is suppressing a strong trait distorter (high k).

We used our recursions to examine whether the spread of the suppressor led to the subsequent loss of the trait distorter. As the suppressor increases in frequency, the population sex ratio becomes less biased, and the fitness benefit of further trait distorter suppression is reduced (negative frequency dependence). This means that, when females are singly mated (λ=1), the rise of the suppressor allele towards some nonzero equilibrium frequency does not cause subsequent loss of the trait distorter (D_1), which remains at fixation (Supplementary Figure 3a). When females are multiply mated (λ>1), there is an additional fertility cost of distortion, and so the suppressor continues to spread, to a higher equilibrium frequency, until the trait distorter (D_1) is lost completely from the population (Supplementary Figure 3c).

We also considered the overall consequences of the trait distorter-suppressor dynamics for the sex ratio. For weak trait distorters (low k), suppressors do not spread. Consequently, there is sex ratio distortion, but it is negligible. For trait distorters of intermediate strength (intermediate k, e.g. $k \approx 0.2$), suppressors are still at low population frequency, and so there can be greater sex ratio distortion. For strong trait distorters (high k), suppressors spread to high population frequency, and so there is little (when λ=1) or no (when λ>1) sex ratio distortion. Consequently, the extent that the sex ratio deviates from the individual optimum of equal investment in the sexes: (a) shows a domed relationship with the extent of distortion (k); and (b)

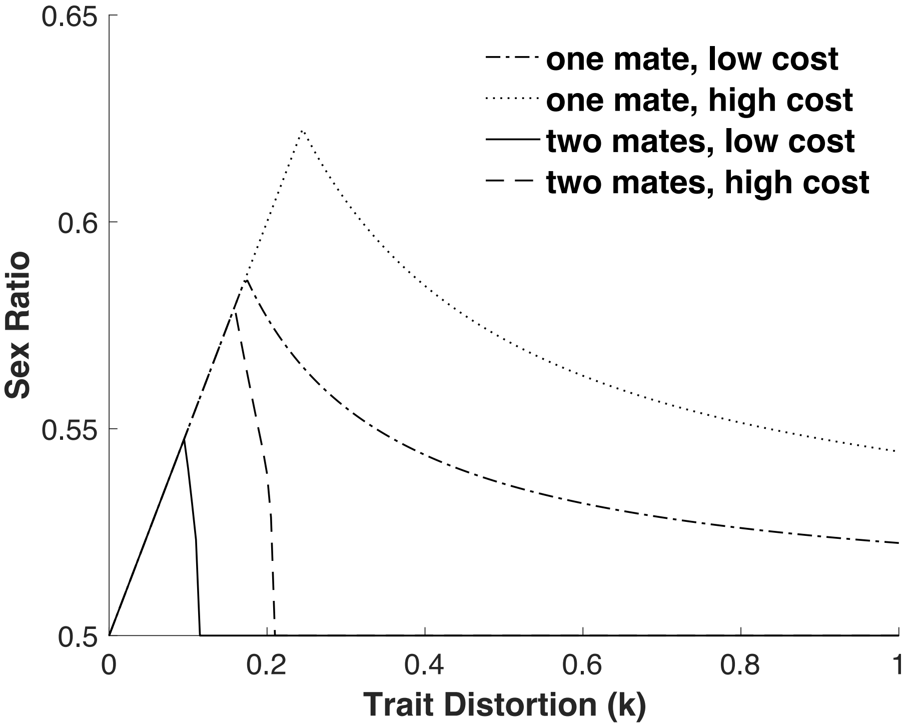

Supplementary Figure 4. Mating frequency and cost of suppression. A sex ratio distorter (D_1) and its suppressor (S_1) are introduced from rarity. Equilibrium sex ratio is plotted, across multiple trails where D_1 has different levels of drive ($0<k\leq1$), and where different assumptions are made about the cost of suppression (c_{sup}) and female mating rate (λ). The four parameter regimes plotted assume single (λ=1) or double (λ=2) female mating; and, low (c_{sup}=0.03) or high (c_{sup}=0.06) viability cost of trait distorter suppression. The sex ratio is more easily distorted when the suppressor is costlier (c_{sup}) and when female mating rate is lower (λ).

will often be negligible[41] (Supplementary Figure 3b & Supplementary Figure 3d). It should be noted that, in reality, the population is a mixture of two types of individual, one adopting a sex ratio of ½ and the other adopting a distorted sex ratio of $(1+k)/2$, and here we are capturing the population average deviation of individuals from the optimal sex ratio.

The case of singly mated females (λ=1) is of special interest because there is no fertility cost of trait distortion ($F_{normal}=F_{drive}$), meaning the individual level cost of

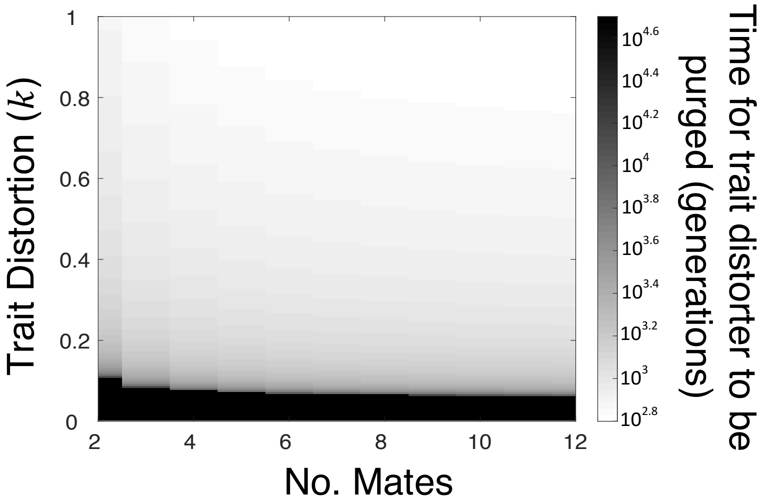

Supplementary Figure 5. Non-equilibrium sex ratio distortion. An X driver (D_1) is introduced from rarity alongside its suppressor (S_1). The proportion of Y-bearing sperm killed by the X driver (k) is varied alongside number of times each female mates per generation (λ). The cost of suppression is fixed at c_{sup}=0.03. We consider trait distorters that are purged from the population, after being suppressed, at equilibrium (λ>1). We plot the number of generations (on a $\log_{10}$ scale) until equilibrium is reached (trials that did not equilibrate by 50,000 generations were capped). We see that stronger trait distorters (high k) are purged at a faster rate, reducing the potential for non-equilibrium sex ratio distortion. Increased female mating (high λ) increases the fertility cost of distortion, meaning trait distorters are purged at a faster rate.

bearing the selfish genetic element (D_1) arises *solely* because an individual level trait (sex ratio) is suboptimal (not ½). Sex ratio distortion is often negligible even in this special case (λ=1), indicating that the parliament of genes can act for the sole purpose of trait (sex ratio) restoration, without the additional incentive of fertility recovery.

Additionally, we considered the effects of model parameters on sex ratio. We found that increasing the rate of female mating (λ) and decreasing the cost of suppression

(c_{sup}) both led to a reduced tolerance of drive, and a correspondingly reduced level of sex ratio distortion (Supplementary Figure 4).

Finally, we considered how far sex ratio can be distorted in the time period after the trait distorter initially invades and before the trait distorter is suppressed and purged from the population. We iterated our recursions and timed how many generations it took to reach equilibrium. We found that stronger trait distorters (higher k) are suppressed and purged from the population more quickly, especially at higher female mating rates (λ) where the fertility cost of sex ratio distortion is greater (Supplementary Figure 5).

4) Evolution of trait distortion

In the above analyses, we assumed that the strength of the trait distorter (D_1) was a fixed parameter (k). We now consider the consequence of allowing the level of trait distortion to evolve[20]. We first consider the scenario in which there is no suppressor. We take a game theoretical approach to find the evolutionarily stable strength of X chromosome sex ratio distortion (k^*) in the absence of suppression. We assume a population where all males have an X chromosome with the same strength of distortion, denoted by a capital K. We then assume that a mutation arises in the X chromosome of one male in the population that causes it to assume a new strength of distortion, denoted by $\check{k}$. We wish to find the strength of distortion that, when adopted by every X chromosome in the population, cannot be invaded by the mutant X chromosome adopting a different strength of distortion. This strength of distortion (k^*) represents the evolutionarily stable strategy (ESS)[44].

Trait distorters have no effect in females, so the fitness of the mutant trait distorter depends only on its action in males. The male bearing the mutant trait distorter has fertility given by its proportional sperm contribution to a female mate's sperm store: $\left(\frac{\left(1-\check{k}/2\right)}{\left(1-\check{k}/2\right)+(\lambda-1)\left(1-K/2\right)}\right)$. The mutant trait distorter is passed into (1+$\check{k}$)/2 offspring, and so the fitness of the mutant X chromosome is proportional to: $w = \frac{(1+\check{k})}{2}\left(\frac{\left(1-\check{k}/2\right)}{\left(1-\check{k}/2\right)+(\lambda-1)\left(1-K/2\right)}\right)$. The ESS strength of X chromosome distortion is the value of k^* that satisfies $\frac{dw}{d\check{k}} = 0$ and $\frac{d^2w}{d\check{k}^2} < 0$ when $\check{k}$=K=k^*, and is given by:

$$k^*=(\lambda+1)/(2\lambda-1). \qquad (5)$$

When females mate singly (λ=1) or doubly (λ=2), maximal trait distorter strength is favoured (k^*=1), resulting in population collapse due to lack of males. As female mating frequency increases to λ≥3, the increased fertility cost of distortion means that the equilibrium strength of distortion (k^*) decreases[20], until it plateaus at the minimum of k^*=0.5 as λ→∞ (Supplementary Figure 6a). The game theoretic equilibrium is verified in fully dynamical population genetic and agent-based simulation models, as described below (Supplementary Figure 6a).

We now consider what sex ratio will evolve in the presence of a suppressor (S_1). We assume that a mutant X chromosome trait distorter (D_2) arises from a mutation on the old sex ratio distorter (D_1), and kills a different proportion of sperm when unsuppressed ($\hat{k}$≠k), biasing individual sex ratio by (1+$\hat{k}$)/2 and reducing ejaculate

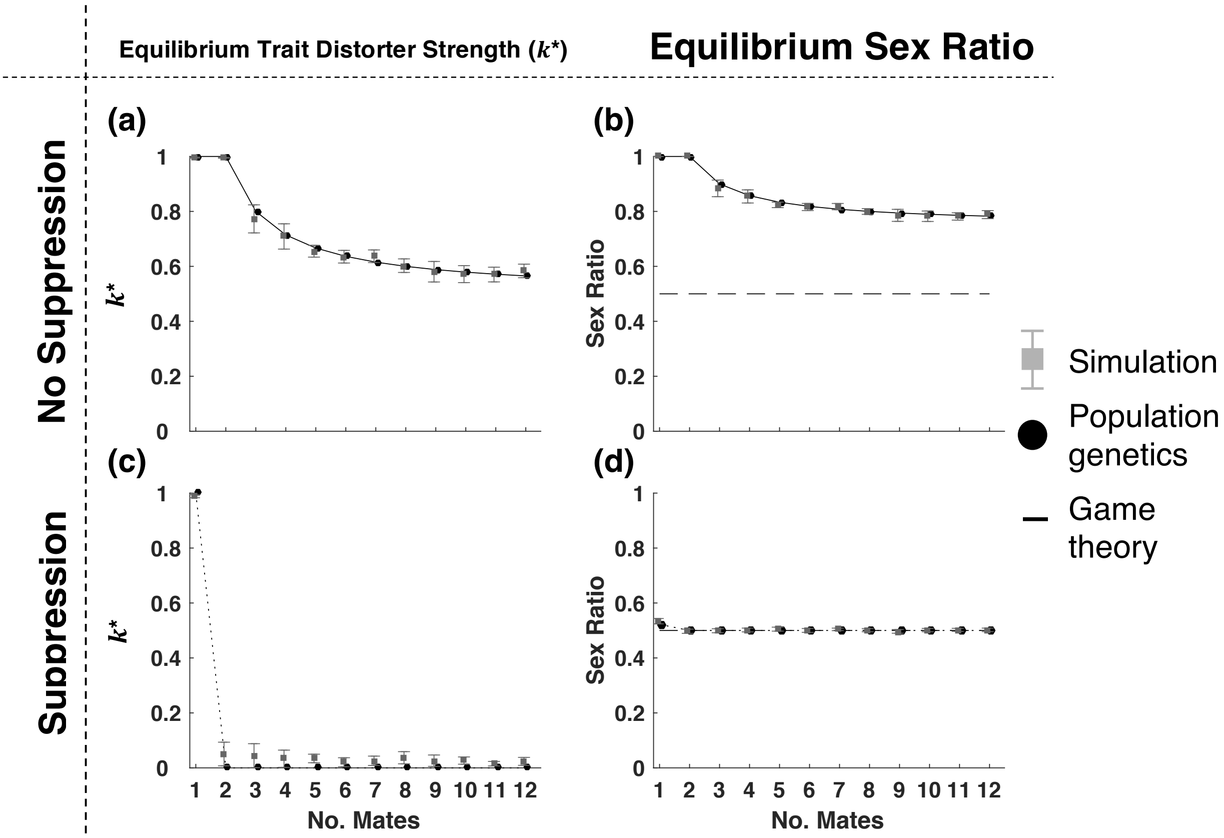

Supplementary Figure 6. Equilibrium trait distorter strength and sex ratio. (a) In the absence of a suppressor (S_1), equilibrium trait distorter strength (k*) decreases with the number of mates each female has per generation (λ). (b) Trait distorters causes more significantly distorted equilibrium sex ratio at lower female mating rates. A dashed line shows the sex ratio that would evolve in the absence of selfish genetic elements (½). (c) In the presence of suppression, maximally distorting (but largely suppressed) trait distorters (k*=1) evolve when females are singly mated (λ=1); otherwise, non-trait distorters (k*=0) evolve. (d) Owing to the spread of suppressors, sex ratio is completely (λ>1) or partially (λ=1) recovered at equilibrium. (c) and (d) assumed a small cost of suppression (c_{sup}=0.03). For all graphs, the results of the simulation (grey boxes) are plotted alongside the population genetics result (black circles). For (a) and (b), the result of a game theoretic analysis is also plotted (solid line). All methods give the same equilibrium trait distorter strength and sex ratio. The error bars show one standard deviation from the mean over 10 trials of the simulation.

size to 1-$\hat{k}$/2. We assume that D_2 and D_1 share a similar genetic and mechanistic basis of drive, such that the mutant distorting X chromosome (D_2) is suppressed by

the same suppressor allele (S_1)[45-48]. In Supplementary Table 4, we display 27 recursions to describe the generational changes in genotype frequencies when the alleles D_1, D_0, D_2, Y, S_0 and S_1 are segregating in a population (notation defined in Supplementary Tables 1 & 3). We note that, in the absence of the trait distorters (D_1 and D_2), population sex ratio evolves to 0.5, and after this, genotype frequencies remain constant over time (Hardy-Weinberg equilibrium). These equations reduce to those in Supplementary Table 2 when genotypes bearing D_2 are set to zero.

We work out the evolved level of sex ratio distortion, under the assumption that trait distorter strength is initially low, and the additional assumption of weak selection. We assume the mutant trait distorter (D_2) is only slightly stronger than the trait distorter from which it is derived (D_1), so that $\hat{k}=k+\delta$, where δ is positive and very small (δ-*weak selection*[49]). We see if a mutant trait distorter can spread by iterating our recursions in Supplementary Table 4 until equilibrium is reached. If the stronger trait distorter (D_2) displaces the weaker one (D_1), we introduce a further mutant trait distorter and iterate our equations again. We elucidate the equilibrium trait distorter strength (k^*) by successively introducing mutant trait distorters (D_2) until one fails to invade, at which point the equilibrium strength (k^*) has been reached.

We find that, in the presence of the suppressor allele (S_1), weakly distorting X chromosomes (low-k) can evade suppression and successfully distort sex ratio. These weak trait distorters will be displaced by slightly more distorting mutants (D_2). If the cost of suppression (c_{sup}) is sufficiently low, this displacement causes the frequency of the suppressor allele (S_1) to increase in response. This trend means

Supplementary Table 3: Further Selection coefficients, drive values, and genotype frequency notation. For each male and female genotype, its proportion in the population at generation *t*, and its probability of maturing from a zygote to an adult (viability, *V*) is given. For each male genotype, the proportion of X chromosomes in its sperm store (drive), and its probability of successfully fertilising the female's egg cell after copulation (fertility, *F*), is given. Male fertility (*F*) depends on the number of mates each female has per generation (λ) according to:

$$F_{normal} = \sum_{i=0}^{\lambda-1} \sum_{j=0}^{\lambda-i-1} \frac{1}{1+\left(1-\frac{k}{2}\right)i+j+(\lambda-i-j-1)(1-\frac{\hat{k}}{2})} l^i (1-l-n)^j n^{\lambda-i-j-1},$$

$$F_{drive} = \sum_{i=0}^{\lambda-1} \sum_{j=0}^{\lambda-i-1} \frac{1-\frac{k}{2}}{\left(1-\frac{k}{2}\right)(i+1)+j+(\lambda-i-j-1)(1-\frac{\hat{k}}{2})} l(t)^i (1-l-n)^j n^{\lambda-i-j-1},$$

$$F_{driveMut} = \sum_{i=0}^{\lambda-1} \sum_{j=0}^{\lambda-i-1} \frac{1-\frac{\hat{k}}{2}}{\left(1-\frac{k}{2}\right)i+j+(\lambda-i-j)(1-\frac{\hat{k}}{2})} l^i (1-l-n)^j n^{\lambda-i-j-1}.$$

l, *n*, and *1-l-n*, are, respectively, the proportions of males in the population with: an unsuppressed D_1; an unsuppressed D_2; neither of these (all other males). k and $\hat{k}$ respectively give the proportion of a male's Y bearing sperm that are killed by an unsuppressed D_1 and D_2 trait distorter, and c_{sup} gives the viability cost of trait distorter suppression.

		Females		Males	
		D_0/D_2	D_1/D_2	D_2/D_2	D_2/Y
S_1 / S_1	***Proportion***	p_{f10}	p_{f13}	p_{f16}	p_{m7}
	Fertility, F	/	/	/	F_{normal}
	Viability, V	1	1	1	1-c_{sup}
	Drive	/	/	/	0.5
S_1 / S_0	***Proportion***	p_{f11}	p_{f14}	p_{f17}	p_{m8}
	Fertility, F	/	/	/	F_{normal}
	Viability, V	1	1	1	1-c_{sup}
	Drive	/	/	/	0.5
S_0 / S_0	***Proportion***	p_{f12}	P_{f15}	p_{f18}	p_{m9}
	Fertility, F	/	/	/	$F_{driveMut}$
	Viability, V	1	1	1	1
	Drive	/	/	/	$(1+\hat{k})/2$

Supplementary Table 4: Recursions detailing the change in proportion of each genotype across one generation (D_0, D_1 and D_2 segregating at trait locus). Notation is defined in Supplementary Table 1 & S3. T is the sum of the right sides of the system of equations such that ∑p=1. It normalises the recursions to ensure that gene frequency changes reflect proportions.

$T\,p_{f1}'$ =	$(p_{f1} + 0.5\,p_{f10} + 0.25\,p_{f11} + 0.5\,p_{f2} + 0.5\,p_{f4} + 0.25\,p_{f5})\,(0.5\,p_{m1} + 0.25\,p_{m2})\,F_{normal}$
$T\,p_{f2}'$ =	$((0.25\,p_{f11} + 0.5\,p_{f12} + 0.5\,p_{f2} + p_{f3} + 0.25\,p_{f5} + 0.5\,p_{f6})\,(0.5\,p_{m1} + 0.25\,p_{m2}) + (p_{f1} + 0.5\,p_{f10} + 0.25\,p_{f11} + 0.5\,p_{f2} + 0.5\,p_{f4} + 0.25\,p_{f5})\,(0.25\,p_{m2} + 0.5\,p_{m3}))\,F_{normal}$
$T\,p_{f3}'$ =	$(0.25\,p_{f11} + 0.5\,p_{f12} + 0.5\,p_{f2} + p_{f3} + 0.25\,p_{f5} + 0.5\,p_{f6})\,(0.25\,p_{m2} + 0.5\,p_{m3})\,F_{normal}$
$T\,p_{f4}'$ =	$(0.5\,p_{f13} + 0.25\,p_{f14} + 0.5\,p_{f4} + 0.25\,p_{f5} + p_{f7} + 0.5\,p_{f8})\,(0.5\,p_{m1} + 0.25\,p_{m2})\,F_{normal} + 1/4\,(p_{f1} + 0.5\,p_{f10} + 0.25\,p_{f11} + 0.5\,p_{f2} + 0.5\,p_{f4} + 0.25\,p_{f5})\,(2\,p_{m4}\,F_{drive} + p_{m5}\,F_{normal})$
$T\,p_{f5}'$ =	$(0.25\,p_{f14} + 0.5\,p_{f15} + 0.25\,p_{f5} + 0.5\,p_{f6} + 0.5\,p_{f8} + p_{f9})\,(0.5\,p_{m1} + 0.25\,p_{m2})\,F_{normal} + (0.5\,p_{f13} + 0.25\,p_{f14} + 0.5\,p_{f4} + 0.25\,p_{f5} + p_{f7} + 0.5\,p_{f8})\,(0.25\,p_{m2} + 0.5\,p_{m3})\,F_{normal} + 1/4\,(0.25\,p_{f11} + 0.5\,p_{f12} + 0.5\,p_{f2} + p_{f3} + 0.25\,p_{f5} + 0.5\,p_{f6})\,(2\,p_{m4}\,F_{drive} + p_{m5}\,F_{drive}) + 1/4\,(p_{f1} + 0.5\,p_{f10} + 0.25\,p_{f11} + 0.5\,p_{f2} + 0.5\,p_{f4} + 0.25\,p_{f5})\,(-2\,(1 + k)\,F_{drive}\,p_{m6} + p_{m5}\,F_{normal})$
$T\,p_{f6}'$ =	$(0.25\,p_{f14} + 0.5\,p_{f15} + 0.25\,p_{f5} + 0.5\,p_{f6} + 0.5\,p_{f8} + p_{f9})\,(0.25\,p_{m2} + 0.5\,p_{m3})\,F_{normal} + 1/4\,(0.25\,p_{f11} + 0.5\,p_{f12} + 0.5\,p_{f2} + p_{f3} + 0.25\,p_{f5} + 0.5\,p_{f6})\,(-2\,(1 + k)\,F_{drive}\,p_{m6} + p_{m5}\,F_{normal})$
$T\,p_{f7}'$ =	$1/4\,(0.5\,p_{f13} + 0.25\,p_{f14} + 0.5\,p_{f4} + 0.25\,p_{f5} + p_{f7} + 0.5\,p_{f8})\,(2\,p_{m4}\,F_{drive} + p_{m5}\,F_{normal})$
$T\,p_{f8}'$ =	$1/4\,((0.25\,p_{f14} + 0.5\,p_{f15} + 0.25\,p_{f5} + 0.5\,p_{f6} + 0.5\,p_{f8} + p_{f9})\,(2\,p_{m4}\,F_{drive} + p_{m5}\,F_{normal}) + (0.5\,p_{f13} + 0.25\,p_{f14} + 0.5\,p_{f4} + 0.25\,p_{f5} + p_{f7} + 0.5\,p_{f8})\,(-2\,(1 + k)\,F_{drive}\,p_{m6} + p_{m5}\,F_{normal}))$
$T\,p_{f9}'$ =	$1/4\,(0.25\,p_{f14} + 0.5\,p_{f15} + 0.25\,p_{f5} + 0.5\,p_{f6} + 0.5\,p_{f8} + p_{f9})\,(-2\,(1 + k)\,F_{drive}\,p_{m6} + p_{m5}\,F_{normal})$
$T\,p_{f10}'$ =	$(0.5\,p_{f10} + 0.25\,p_{f11} + 0.5\,p_{f13} + 0.25\,p_{f14} + p_{f16} + 0.5\,p_{f17})\,(0.5\,p_{m1} + 0.25\,p_{m2})\,F_{normal} - 1/4\,(p_{f1} + 0.5\,p_{f10} + 0.25\,p_{f11} + 0.5\,p_{f2} + 0.5\,p_{f4} + 0.25\,p_{f5})\,(-2\,p_{m7} - p_{m8})\,F_{driveMut}$
$T\,p_{f11}'$ =	$((0.25\,p_{f11} + 0.5\,p_{f12} + 0.25\,p_{f14} + 0.5\,p_{f15} + 0.5\,p_{f17} + p_{f18})\,(0.5\,p_{m1} + 0.25\,p_{m2}) + (0.5\,p_{f10} + 0.25\,p_{f11} + 0.5\,p_{f13} + 0.25\,p_{f14} + p_{f16} + 0.5\,p_{f17})\,(0.25\,p_{m2} + 0.5\,p_{m3}))\,F_{normal}$
$T\,p_{f12}'$ =	$(0.25\,p_{f11} + 0.5\,p_{f12} + 0.25\,p_{f14} + 0.5\,p_{f15} + 0.5\,p_{f17} + p_{f18})\,(0.25\,p_{m2} + 0.5\,p_{m3})\,F_{normal} + 1/4\,(0.25\,p_{f11} + 0.5\,p_{f12} + 0.5\,p_{f2} + p_{f3} + 0.25\,p_{f5} + 0.5\,p_{f6})\,(-2\,(1 + \hat{k})\,F_{driveMut}\,p_{m9} + p_{m8}\,F_{driveMut})$
$T\,p_{f13}'$ =	$1/4\,((0.5\,p_{f10} + 0.25\,p_{f11} + 0.5\,p_{f13} + 0.25\,p_{f14} + p_{f16} + 0.5\,p_{f17})\,(2\,p_{m4}\,F_{drive} + p_{m5}\,F_{normal}) - (0.5\,p_{f13} + 0.25\,p_{f14} + 0.5\,p_{f4} + 0.25\,p_{f5} + p_{f7} + 0.5\,p_{f8})\,(-2\,p_{m7} - p_{m8})\,F_{driveMut})$
$T\,p_{f14}'$ =	$1/4\,((0.25\,p_{f11} + 0.5\,p_{f12} + 0.25\,p_{f14} + 0.5\,p_{f15} + 0.5\,p_{f17} + p_{f18})\,(2\,p_{m4}\,F_{drive} + p_{m5}\,F_{normal}) + (0.5\,p_{f10} + 0.25\,p_{f11} + 0.5\,p_{f13} + 0.25\,p_{f14} + p_{f16} + 0.5\,p_{f17})\,(-2\,(1 + k)\,F_{drive}\,p_{m6} + p_{m5}\,F_{normal}) + (0.5\,p_{f13} + 0.25\,p_{f14} + 0.5\,p_{f4} + 0.25\,p_{f5} + p_{f7} + 0.5\,p_{f8})\,(-2\,(1 + \hat{k})\,F_{driveMut}\,p_{m9} + p_{m8}\,F_{driveMut}) - (0.25\,p_{f14} + 0.5\,p_{f15} + 0.25\,p_{f5} + 0.5\,p_{f6} + 0.5\,p_{f8} + p_{f9})\,(-2\,p_{m7} - p_{m8})\,F_{driveMut})$
$T\,p_{f15}'$ =	$1/4\,((0.25\,p_{f11} + 0.5\,p_{f12} + 0.25\,p_{f14} + 0.5\,p_{f15} + 0.5\,p_{f17} + p_{f18})\,(-2\,(1 + k)\,F_{drive}\,p_{m6} + p_{m5}\,F_{normal}) + (0.25\,p_{f14} + 0.5\,p_{f15} + 0.25\,p_{f5} + 0.5\,p_{f6} + 0.5\,p_{f8} + p_{f9})\,(-2\,(1 + \hat{k})\,F_{driveMut}\,p_{m9} + p_{m8}\,F_{driveMut}))$
$T\,p_{f16}'$ =	$1/4\,(0.5\,p_{f10} + 0.25\,p_{f11} + 0.5\,p_{f13} + 0.25\,p_{f14} + p_{f16} + 0.5\,p_{f17})\,(-2\,p_{m7} - p_{m8})\,F_{driveMut}$
$T\,p_{f17}'$ =	$1/4\,((0.5\,p_{f10} + 0.25\,p_{f11} + 0.5\,p_{f13} + 0.25\,p_{f14} + p_{f16} + 0.5\,p_{f17})\,(-2\,(1 + \hat{k})\,F_{driveMut}\,p_{m9} + p_{m8}\,F_{driveMut}) - (0.25\,p_{f11} + 0.5\,p_{f12} + 0.25\,p_{f14} + 0.5\,p_{f15} + 0.5\,p_{f17} + p_{f18})\,(-2\,p_{m7} - p_{m8})\,F_{driveMut})$
$T\,p_{f18}'$ =	$1/4\,(0.25\,p_{f11} + 0.5\,p_{f12} + 0.25\,p_{f14} + 0.5\,p_{f15} + 0.5\,p_{f17} + p_{f18})\,(-2\,(1 + \hat{k})\,F_{driveMut}\,p_{m9} + p_{m8}\,F_{driveMut})$
$T\,p_{m1}'$ =	$1/4\,(p_{f1} + 0.5\,p_{f10} + 0.25\,p_{f11} + 0.5\,p_{f2} + 0.5\,p_{f4} + 0.25\,p_{f5})\,((2\,p_{m1} + p_{m2})\,F_{normal} - 2\,F_{drive}\,p_{m4} + p_{m5}\,F_{normal} + 2\,p_{m7}\,F_{driveMut} + p_{m8}\,F_{driveMut})$
$T\,p_{m2}'$ =	$1/4\,((p_{f1} + 0.5\,p_{f10} + 0.25\,p_{f11} + 0.5\,p_{f2} + 0.5\,p_{f4} + 0.25\,p_{f5})\,((p_{m2} + 2\,p_{m3})\,F_{normal} + 2\,(-1 + k)\,F_{drive}\,p_{m6} + 2\,(-1 + \hat{k})\,F_{driveMut}\,p_{m9} + p_{m5}\,F_{normal} + p_{m8}\,F_{driveMut}) + (0.25\,p_{f11} +$

	$0.5 p_{f12} + 0.5 p_{f2} + p_{f3} + 0.25 p_{f5} + 0.5 p_{f6}) ((2 p_{m1} + p_{m2}) F_{normal} - 2 F_{drive} p_{m4} + p_{m5} F_{normal} + 2 p_{m7} F_{driveMut} + p_{m8} F_{driveMut}))$
T p_{m3}' =	$1/4 (0.25 p_{f11} + 0.5 p_{f12} + 0.5 p_{f2} + p_{f3} + 0.25 p_{f5} + 0.5 p_{f6}) ((p_{m2} + 2 p_{m3}) F_{normal} + 2 (-1 + k) F_{drive} p_{m6} + 2 (-1 + \hat{k}) F_{driveMut} p_{m9} + p_{m5} F_{normal} + p_{m8} F_{driveMut})$
T p_{m4}' =	$1/4 V_{suppression} (0.5 p_{f13} + 0.25 p_{f14} + 0.5 p_{f4} + 0.25 p_{f5} + p_{f7} + 0.5 p_{f8}) ((2 p_{m1} + p_{m2}) F_{normal} - 2 F_{drive} p_{m4} + p_{m5} F_{normal} + 2 p_{m7} F_{driveMut} + p_{m8} F_{driveMut})$
T p_{m5}' =	$1/4 V_{suppression} ((0.5 p_{f13} + 0.25 p_{f14} + 0.5 p_{f4} + 0.25 p_{f5} + p_{f7} + 0.5 p_{f8}) ((p_{m2} + 2 p_{m3}) F_{normal} + 2 (-1 + k) F_{drive} p_{m6} + 2 (-1 + \hat{k}) F_{driveMut} p_{m9} + p_{m5} F_{normal} + p_{m8} F_{driveMut}) + (0.25 p_{f14} + 0.5 p_{f15} + 0.25 p_{f5} + 0.5 p_{f6} + 0.5 p_{f8} + p_{f9}) ((2 p_{m1} + p_{m2}) F_{normal} - 2 F_{drive} p_{m4} + p_{m5} F_{normal} + 2 p_{m7} F_{driveMut} + p_{m8} F_{driveMut}))$
T p_{m6}' =	$1/4 (0.25 p_{f14} + 0.5 p_{f15} + 0.25 p_{f5} + 0.5 p_{f6} + 0.5 p_{f8} + p_{f9}) ((p_{m2} + 2 p_{m3}) F_{normal} + 2 (-1 + k) F_{drive} p_{m6} + 2 (-1 + \hat{k}) F_{driveMut} p_{m9} + p_{m5} F_{normal} + p_{m8} F_{driveMut})$
T p_{m7}' =	$1/4 V_{suppression} (0.5 p_{f10} + 0.25 p_{f11} + 0.5 p_{f13} + 0.25 p_{f14} + p_{f16} + 0.5 p_{f17}) ((2 p_{m1} + p_{m2}) F_{normal} - 2 F_{drive} p_{m4} + p_{m5} F_{normal} + 2 p_{m7} F_{driveMut} + p_{m8} F_{driveMut})$
T p_{m8}' =	$1/4 V_{suppression} ((0.5 p_{f10} + 0.25 p_{f11} + 0.5 p_{f13} + 0.25 p_{f14} + p_{f16} + 0.5 p_{f17}) ((p_{m2} + 2 p_{m3}) F_{normal} + 2 (-1 + k) F_{drive} p_{m6} + 2 (-1 + \hat{k}) F_{driveMut} p_{m9} + p_{m5} F_{normal} + p_{m8} F_{driveMut}) + (0.25 p_{f11} + 0.5 p_{f12} + 0.25 p_{f14} + 0.5 p_{f15} + 0.5 p_{f17} + p_{f18}) ((2 p_{m1} + p_{m2}) F_{normal} - 2 F_{drive} p_{m4} + p_{m5} F_{normal} + 2 p_{m7} F_{driveMut} + p_{m8} F_{driveMut}))$
T p_{m9}' =	$1/4 (0.25 p_{f11} + 0.5 p_{f12} + 0.25 p_{f14} + 0.5 p_{f15} + 0.5 p_{f17} + p_{f18}) ((p_{m2} + 2 p_{m3}) F_{normal} + 2 (-1 + k) F_{drive} p_{m6} + 2 (-1 + \hat{k}) F_{driveMut} p_{m9} + p_{m5} F_{normal} + p_{m8} F_{driveMut})$

that, given sequential mutations on the X chromosome to increase sex ratio distortion, suppression will ultimately evolve, completely ($\lambda>1$) or partially ($\lambda=1$) restoring an equal sex ratio (Figure 3d & Supplementary Figure 6c & Supplementary Figure 6d). Consequently, we conclude that, with reasonable assumptions about the cost of suppression (c_{sup}), trait distorter suppression is the ultimate outcome of trait distorter evolution. For the sex ratio to be appreciably distorted (>60% females), suppression cost needs to exceed around c_{sup}=0.15 ($\lambda=1$) or c_{sup}=0.35 ($\lambda>1$) (Supplementary Figure 8).

By setting the frequencies of all genotypes bearing the suppressor (S_1) to zero, we can use our recursions in Supplementary Table 4 to find the equilibrium strength of trait distortion in the absence of suppression. We exactly recover the equilibrium derived in Supplementary Equation 5, which gives the ESS strength of X

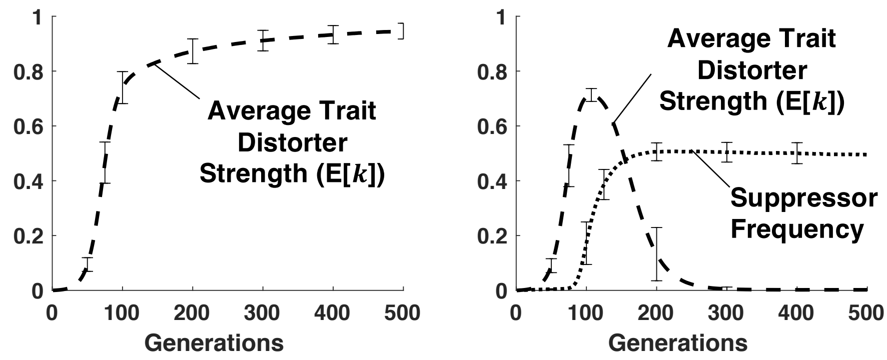

Supplementary Figure 7: Evolution of sex ratio distortion (dynamics). This figure plots the results of the agent-based simulation model, in which X chromosome drive can mutate and take any value within 0-1. In (a), there is no suppressor allele (S_1), and the population average level of X chromosome drive (E[k]) tends towards maximum strength (k*=1). In (b), a suppressor of distortion (S_1) is introduced from rarity. The population average strength of X chromosome distortion (E[k]) increases alongside the suppressor (S_1) frequency. Eventually, a threshold is passed, after which, distorting X chromosomes ($k_a,k_b>0$) are lost from the population. Sex ratio is restored to 0.5 at equilibrium. Double female mating (λ=2) and high suppression cost (c_{sup}=0.3) were assumed in these simulations. The plots show average values over 100 runs, for N=100,000 individuals, with error bars plotting one standard deviation in each direction of the mean.

chromosome trait distortion (k*) in the absence of suppression (Supplementary Figure 6a).

Agent-based simulation

We construct an agent-based simulation to ask what level of sex ratio distortion evolves under strong selection, and when continuous variation is permitted at trait distorter and suppressor loci. We model a population of N=10,000 individuals and track evolution at an X chromosome trait distorter locus and an autosomal suppressor locus. Individuals either have two alleles at the X chromosome

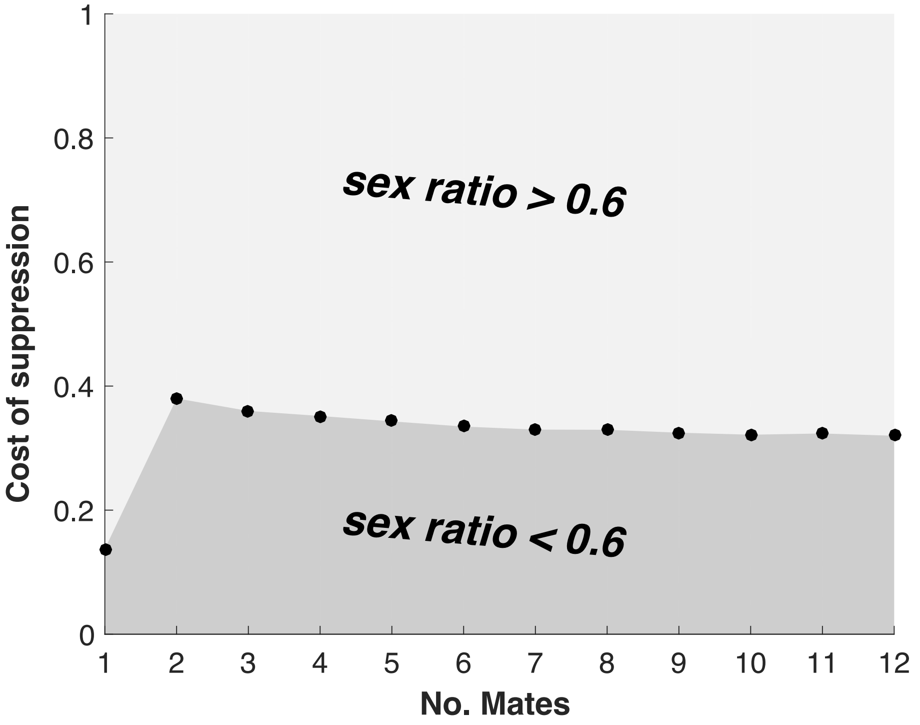

Supplementary Figure 8. Cost of suppression required for appreciable sex ratio distortion. The equilibrium strength of X chromosome distortion was obtained for different costs of suppression (c_{sup}). The sex ratio at this equilibrium level of X chromosome was recorded. The cost of suppression required for sex ratio distortion to be appreciably distorted (>60% females produced) is plotted (solid circles). Sex ratio is significantly distorted for the region above this curve.

locus, with strengths denoted by k_a and k_b (females), or one allele at the X chromosome locus, with strength denoted by k_a (males), and one Y chromosome.

Each allele at the trait distorter locus can take any continuous value between zero and one. Individuals have two alleles at the suppressor locus, with strengths denoted by m_a and m_b (diploid). At the suppressor locus, we consider both the case of: (i) discrete variation, in which suppressor strengths are either zero or one, and (ii) continuous variation, in which suppressor strengths can take any continuous value between zero and one. We assume that the strongest (highest value)

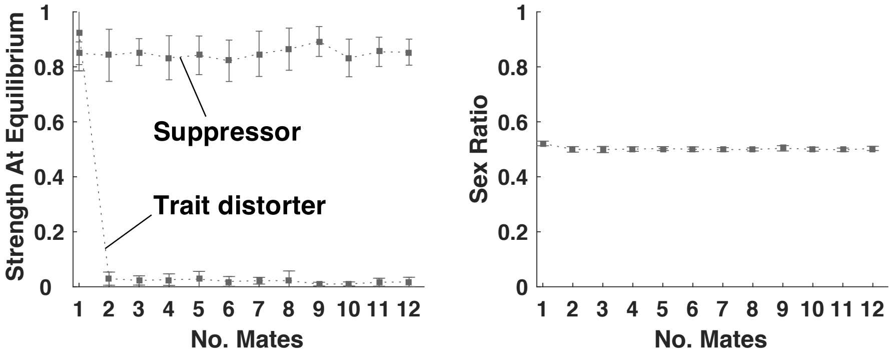

Supplementary Figure 9. Equilibrium trait distorter strength and sex ratio under continuous suppressor variation. (a) Equilibrium suppressor strength (E[m*]) evolves to be high (~0.83). Maximally distorting (but largely suppressed) X chromosomes (E[k*]=1) evolve when females are singly mated (λ=1); otherwise, non-distorting X chromosomes (E[k*]=0) evolve. (b) Owing to the evolution of strong suppression, sex ratio is completely (λ>1) or partially (λ=1) recovered at equilibrium. Suppression cost in these simulations was c_{sup}=0.03. Error bars show one standard deviation from the mean over 10 trials.

suppressor allele within an individual is dominant. The ejaculate size of a given male is $1 - \frac{(1-\max(m_a, m_b))k_a}{2}$, and his fertility is this value divided by the total sperm stored in the females it mates with. The total sperm store is the sum of ejaculates of this male and λ other males drawn at random from the population with replacement. The viability of males with an active trait distorter (k_a>0) is given by 1-max(m_a,m_b)c_{sup}. The viability of all other individuals is 1.

Each generation, there are N breeding pairs. Females are drawn at random with replacement to fill each female position in each breeding pair. Males are drawn from the population, with replacement, with probabilities given by their fertility. Breeding pairs then reproduce to produce one offspring, before dying (non-overlapping generations). Alleles at the suppressor locus are inherited in Mendelian fashion.

Alleles at the trait distorter locus in males may drive, meaning the X chromosome is inherited, rather than the Y chromosome, with the probability $(1+k_a(1-\max(m_a,m_b)))/2$. Offspring then compete for spots in the adult population, of which there are N. To fill each spot, offspring are drawn with replacement with likelihood that is proportional to their viability. Each generation, alleles at the trait distorter locus, and for the case of continuous variation at the suppressor locus, alleles at the suppressor locus have a 0.0005 chance of mutating to a new value, which is drawn from a normal distribution centred around the pre-mutation value, with variance 0.5, and truncated between 0 and 1 (strong selection). For the case of discrete variation at the suppressor locus, alleles at the suppressor locus have a 0.001 chance of mutating each generation between suppressor and non-suppressor states.

We iterate this lifecycle over 5,000 generations. We see that, when discrete variation is permitted at the suppressor locus, the simulation quantitatively recovers the equilibrium level of distortion, and corresponding sex ratio, given by the game theoretic and population genetic models (Supplementary Figure 6). When continuous variation is permitted at the suppressor locus, qualitatively equivalent results are obtained: suppressor strength ($E[m]$) evolves to be high enough that sex ratio is fully ($\lambda \geq 2$) or partially ($\lambda=1$) recovered at equilibrium (Supplementary Figure 9).

Supplementary Note 4

Genomic Imprinting and Altruism

Genomic imprinting occurs at a minority of genes in mammals and flowering plants. An imprinted allele has different epigenetic marks, and corresponding expression levels, when maternally and paternally inherited[50]. We examined the evolution of an altruistic helping behaviour in a population capable of genomic imprinting. A behaviour is altruistic if it incurs a cost (*c*) to perform, by the actor, and provides a benefit (*b*) to another individual, the recipient. Altruism is favoured if the genetic relatedness (*R*) between the actor and recipient is sufficiently high, such that $Rb>c$[51].

An individual may be more closely related to their social partners via their maternal or paternal genes[52-54]. For example, if a female mates two males, then on average her offspring would be related by $R_m=1/2$ at maternal genes and $R_p=1/4$ at paternal genes. If genes can 'gain information' about where they came from, by imprinting, then they could be selected to adjust traits accordingly. Assume that relatedness to social partners is R_p *and* R_m at paternal and maternal genes respectively. In this case, altruistic helping would be favoured at: maternally imprinted genes when $R_mb>c$; paternally imprinted genes when $R_pb>c$; and unimprinted genes when $((R_p+R_m)/2)b>c$[54-56]. Consequently, if $R_mb>c>((R_p+R_m)/2)b$, then altruistic helping is favoured at maternally imprinted genes, when it is disfavoured at unimprinted genes (selfish trait distortion).

We consider an autosomal, maternally expressed selfish genetic element that may gain a propagation advantage by upregulating individual altruistic investment[55,57-61].

The genes that do not gain a propagation advantage from altruism upregulation comprise both paternally expressed and unimprinted genes. The conflict between maternally and paternally expressed genes, which can result in arms races and a 'tug of war' over organism phenotype, has been considered in previous theoretical work[62-65]. However, we focus on unimprinted suppressors, for simplicity, and because unimprinted genes comprise the larger group of genes, constituting the majority within the parliament of genes[50,66,67]. We focus our analyses on when a maternally expressed trait distorter and an unimprinted suppressor can spread. We first describe our modelling assumptions, then successively analyse the cases of unimprinted, and imprinted, altruism. The purpose of this model is to illustrate how selection will act on selfish imprinted genes and their suppressors.

Modelling Assumptions

We track a large population of diploid individuals. We consider a gene that induces an altruistic investment of some amount (k>0), at a fitness cost to the individual ($c(k)$), which is a monotonically increasing function of altruistic investment $\left(\frac{\partial c}{\partial k} \geq 0\right)$, and a benefit to the social partner ($b(k)$>$c(k)$), which is a function that is monotonically increasing with the level of altruistic investment $\left(\frac{\partial b}{\partial k} \geq 0\right)$ yet diminishing with the *cost* of altruistic investment $\left(\frac{d^2 b}{dc^2} \leq 0\right)$[68].

Each generation, male gametes (e.g. sperm) fuse at random with female gametes (e.g. eggs) to generate individuals (random mating). Individuals then pair up with other individuals who have matching maternally (egg-) inherited alleles at all loci;

pairs are random with respect to identity at the paternally (sperm-) inherited allele at all loci (R_m=1; R_p=0). Individuals may then invest in altruism directed towards their partner, before producing gametes in proportion to their fitness (fertility), and dying (non-overlapping generations).

In nature, relatedness asymmetries within a generation may be generated by sex biased migration patterns[58], or as a consequence of greater variance in reproductive success in males[52,69]. They may alternatively be generated if kin recognition alleles are imprinted, which has been implicated in humans[70] and mice[71-73].

Unimprinted Altruism

We consider an unimprinted altruism gene, denoted by y_A, that, when homozygous, induces an altruistic investment of k_A ($k_A>0$), and when heterozygous, induces an altruistic investment of $h*k_A$, where h denotes the dominance. If we take g and g' as the population frequency of the altruism gene in two consecutive generations, then the population frequency of the altruism gene in the latter generation is:

$$\bar{w}g' = g^2\left(\frac{b(k_A)g(1-g)h}{g^2+g(1-g)} + \frac{b(k_A)g^2}{g^2+g(1-g)} - c(k_A) + 1\right) + \frac{1}{2}g(1-g)(\frac{g(1-g)b(k_A)h}{g^2+g(1-g)} + \frac{b(k_A)g^2}{g^2+g(1-g)} - c(k_A)h + 1) + \frac{1}{2}(1-g)g(\frac{b(k_A)(1-g)gh}{(1-g)^2+(1-g)g} - c(k_A)h + 1), \quad (6)$$

where the mean fitness of individuals is given by: $\bar{w} = g(1-g)\left(\frac{g(1-g)b(k_A)h}{g^2+g(1-g)} + \frac{b(k_A)g^2}{g^2+g(1-g)} - c(k_A)h + 1\right) + g^2\left(\frac{g(1-g)b(k_A)h}{g^2+g(1-g)} + \frac{b(k_A)g^2}{g^2+g(1-g)} - c(k_A) + 1\right) + (1-g)g\left(\frac{(1-g)gb(k_A)h}{(1-g)^2+(1-g)g} - c(k_A)h + 1\right) + (1-g)^2\left(\frac{(1-g)gb(k_A)h}{(1-g)^2+(1-g)g} + 1\right).$

Each term relates to a different class of individual. For illustration, we derive the term relating to heterozygous individuals with a maternally derived y_A: $\frac{1}{2}g(1-g)\left(\frac{g(1-g)b(k_A)h}{g^2+g(1-g)}+\frac{b(k_A)g^2}{g^2+g(1-g)}-c(k_A)h+1\right)$. (1) Find the frequency of individuals with this genotype: g*(1-g). (2) Multiply this by absolute fitness, which is 1 at baseline, with additively applied benefits weighted by the probability that the individual pairs with altruists, and additively applied costs applied if the individual is an altruist: $\frac{g(1-g)b(k_A)h}{g^2+g(1-g)}+\frac{b(k_A)g^2}{g^2+g(1-g)}-c(k_A)h+1$. (3) Weight this by the proportion of y_A-bearing gametes produced by individuals: ½.

The altruism gene decreases in population frequency when $g' < g$, which requires the condition: ½b(k_A)<c(k_A). Given that genetic relatedness is $(R_f+R_m)/2$ = (0+1)/2 = ½, this condition corresponds to Hamilton's Rule[51,74,75]. This (½b(k_A)<c(k_A)) is also the condition for the invasion of a weaker altruism gene (lower k_A) against a stronger one, owing to diminishing returns on altruistic investment $\left(\frac{d^2b}{dc^2}<0\right)$. Taken together, this implies that, when ½b(k_A)<c(k_A), the optimal altruism investment for unimprinted genes is zero, and increased altruistic investment is increasingly suboptimal.

Trait Distorter Spread

We consider an imprinted altruism gene that is only expressed when maternally inherited, denoted by D_1, and induces an altruistic investment of k (k>0). If we take p and p' as the population frequency of the altruism gene in two consecutive

generations, then the population frequency of the altruism gene in the latter generation is:

$$\bar{w}p' = p(1-p)(b(k)-c(k)+1)/2 + (1-p)p/2 + p^2(b(k)-c(k)+1), \quad (7)$$

where the mean fitness of individuals is given by: $\bar{w} = 1 - p + (b(k) - c(k) + 1)p$.

Each term relates to a different class of individual. For illustration, we derive the term relating to heterozygous individuals with a paternally derived D_1: (1-*p*)*p*/2. (1) Find the population frequency of individuals with this genotype: (1-*p*)**p*. (2) Weight by (absolute) individual fitness: 1. (3) Weight by the proportion of imprinted altruism gene-bearing gametes (D_1) produced: ½.

We ask when a rare imprinted altruism gene (D_1) can invade a population fixed for the non-trait distorter (D_0). We take Supplementary Equation 7, set *p*'=*p*=*p**, and solve to find two possible equilibria: *p**=0 (non-trait distorter fixation) and *p**=1 (imprinted gene fixation). The imprinted gene (D_1) can invade from rarity when the *p**=0 equilibrium is unstable, which occurs when the differential of *p*' with respect to *p*, at *p**=0, is greater than one. The imprinted altruism gene invasion criterion is therefore $b(k)>c(k)$.

We now ask what frequency the imprinted altruism gene (D_1) will reach after invasion. The gene (D_1) can spread to fixation if the *p**=1 equilibrium is stable, which requires that the differential of *p*' with respect to *p*, at p*=1, is less than one. This

requirement always holds true, demonstrating that there is no negative frequency dependence on the imprinted gene, and that it will always spread to fixation after its initial invasion.

Given that genetic relatedness is R_m=1, our condition for the spread of the imprinted altruism gene ($b(k)>c(k)$) corresponds to Hamilton's Rule[51,74,75]. Combining with the result of the "Unimprinted altruism" model, altruistic investment (of $k_I=k_A=k$) is simultaneously favoured at maternally expressed genes and disfavoured at unimprinted genes, rendering the imprinted altruism gene a selfish trait distorter, when $\frac{1}{2}b(k_I)<c(k_I)<b(k_I)$.

Spread of an autosomal suppressor

We ask when an unimprinted suppressor (S_1), competing against a non-suppressor (S_0), will invade from rarity. We can write recursions detailing the generational change in the frequencies of the four possible gametes, D_0/S_0, D_0/S_1, D_1/S_0, D_1/S_1, with the respective frequencies in the current generation denoted by x_{00}, x_{01}, x_{10} and x_{11}, and the frequencies in the subsequent generation denoted by an appended dash ('):

$$\bar{w}x_{00}' = x_{00}x_{00} + \frac{x_{00}x_{01}}{2} + \frac{x_{00}x_{10}}{2} + \frac{x_{00}x_{11}}{4} + \frac{x_{01}x_{00}}{2} + \frac{x_{01}x_{10}}{4} + \frac{1}{4}x_{11}x_{00}(1 - c_{sup}) + \frac{1}{4}x_{10}x_{01}(1 - c_{sup})(1 + b(k)(x_{00} + x_{10})) + \frac{1}{2}x_{10}x_{00}(1 - c(k) + b(k)(x_{00} + x_{10})) \quad (8)$$

$$\bar{w}x_{01}' = \frac{x_{00}}{4} + \frac{x_{00}x_{01}}{2} + \frac{x_{00}x_{11}}{4} + \frac{x_{01}x_{00}}{2} + x_{01}x_{01} + \frac{x_{01}x_{10}}{4} + \frac{x_{01}x_{11}}{2} + \frac{1}{4}x_{11}x_{00}(1 - c_{sup}) + \frac{1}{2}x_{11}x_{01}(1 - c_{sup}) + \frac{1}{4}x_{10}x_{01}(1 - c_{sup})(1 + b(k)(x_{00} + x_{10})) \quad (9)$$

$$\bar{w}x_{10}' = \frac{x_{00}x_{10}}{2} + \frac{x_{00}x_{11}}{4} + \frac{x_{01}x_{10}}{4} + \frac{1}{4}x_{11}x_{00}(1 - c_{sup}) + \frac{1}{2}x_{11}x_{10}(1 - c_{sup}) + \frac{1}{4}x_{10}x_{01}(1 - c_{sup})$$

$$(1 + b(k)(x_{00} + x_{10})) + \frac{1}{2}x_{10}x_{11}(1 - c_{sup})(1 + b(k)(x_{00} + x_{10})) + \frac{1}{2}x_{10}x_{00}(1 -$$

$$c(k) + b(k)(x_{00} + x_{10})) + x_{10}x_{10}(1 - c(k) + b(k)(x_{00} + x_{10})) \qquad (10)$$

$$\bar{w}x_{11}' = \frac{x_{00}x_{11}}{4} + \frac{x_{01}x_{10}}{4} + \frac{x_{01}x_{11}}{2} + \frac{1}{4}x_{11}x_{00}(1 - c_{sup}) + \frac{1}{2}x_{11}x_{01}(1 - c_{sup}) + \frac{1}{2}x_{11}x_{10}(1 - c_{sup})$$

$$+x_{11}x_{11}(1 - c_{sup}) + \frac{1}{4}x_{10}x_{01}(1 - c_{sup})(1 + b(k)(x_{00} + x_{10})) + \frac{1}{2}x_{10}x_{11}(1 - c_{sup})(1$$

$$+b(k)(x_{00} + x_{10})) \qquad (11)$$

$\bar{w}$ is the average fitness of individuals in the current generation, and equals the sum of the equations' right-hand sides. Each term in each equation relates to a different class of individual. For illustration, we derive the term corresponding to the contribution of D_0/S_0 gametes to the next generation, by individuals with a maternally inherited D_1/S_0 gamete and a paternally inherited D_0/S_0 gamete; this is the $\frac{1}{2}x_{10}x_{00}(1 - c(k) + b(k)(x_{00} + x_{10}))$ term in the $\bar{w}x_{00}'$ recursion. (1) Find the population frequency of individuals with this genotype: $x_{10}x_{00}$. (2) Weight by (absolute) individual fitness: $1 - c(k) + b(k)(x_{00} + x_{10})$. (3) Weight by the proportion of trait distorter-bearing gametes (D_1) produced: ½.

We derive the Jacobian stability matrix for the equilibrium in which the trait distorter (D_1) and non-suppressor (S_0) are at fixation ($x_{00}^*=0$, $x_{01}^*=0$, $x_{10}^*=1$, $x_{11}^*=0$). The suppressor can invade when the equilibrium is unstable, which occurs when the leading eigenvalue is greater than one. The leading eigenvalue is $\frac{(b(k)+2)(1-c_{sup})}{2(b(k)-c(k)+1)}$, meaning the suppressor invasion criterion is given by:

$$c_{sup}(1+b(k)/2)<c(k)-b(k)/2. \qquad (12)$$

Therefore, the suppressor invades from rarity above a threshold level of distortion, k, when, from the perspective of an unimprinted locus, the number of relatives that die as a result of trait distortion ($c(k)$-$b(k)/2$), exceeds the number of relatives that die as a result of trait distorter suppression ($c_{sup}(1+b(k)/2)$).

Consequences of suppressor spread for organism phenotype

We ask what frequency the trait distorter (D_1) and suppressor (S_1) will reach after initial suppressor (S_1) invasion. We assume that the suppressor is introduced from rarity when the trait distorter has reached the population frequency given by f ($x_{00}\rightarrow f$, $x_{10}\rightarrow 1-f$, $\{x_{01},x_{11}\}\rightarrow 0$). We numerically iterate Supplementary Equations 8-11, over successive generations, until equilibrium has been reached. At equilibrium, for all parameter combinations (f,t,c_{sup},c_{trait}), the suppressor reaches an internal equilibrium and the trait distorter is lost from the population ($x_{00}^*+x_{01}^*=1$, $x_{10}^*=0$, $x_{11}^*=0$). This equilibrium arises because trait distorter-presence gives the suppressor (S_1) a selective advantage, leading to high suppressor frequency, which in turn reverses the selective advantage of the trait distorter (D_1), leading to trait distorter loss and suppressor equilibration (Figure 3b).

Invasion of a mutant trait distorter

We ask when a mutant trait distorter (D_2) of strength $\hat{k}$ will invade against a resident trait distorter (D_1) that is unsuppressed and at fixation ($\hat{k}\neq k$). We write recursions detailing the generational frequency changes in the six possible gametes, D_0/S_0, D_0/S_1, D_1/S_0, D_1/S_1, D_2/S_0, D_2/S_1, with current generation frequencies denoted

respectively by x_{00}, x_{01}, x_{10}, x_{11}, x_{20}, x_{21}, and next generation frequencies denoted with an appended dash ('):

$$\bar{w}x_{00}' = x_{00}x_{00} + \frac{x_{00}x_{01}}{2} + \frac{x_{00}x_{10}}{2} + \frac{x_{00}x_{11}}{4} + \frac{x_{00}x_{20}}{2} + \frac{x_{00}x_{21}}{4} + \frac{x_{02}x_{00}}{2} + \frac{x_{01}x_{10}}{4} + \frac{x_{01}x_{20}}{4} + \frac{1}{4}x_{11}x_{00}\left(1 - c_{sup}\right) + \frac{1}{4}x_{21}x_{00}(1 - c_{sup}) + \frac{1}{4}x_{10}x_{01}(1 - c_{sup})(1 + b(k)(x_{00} + x_{10} + x_{20})) + \frac{1}{2}x_{10}x_{00}(1 - c(k) + b(k)(x_{00} + x_{10} + x_{20})) + \frac{1}{4}x_{20}x_{01}(1 - c_{sup})(1 + b(\hat{k})(x_{00} + x_{10} + x_{20})) + \frac{1}{2}x_{20}x_{00}(1 - c(\hat{k}) + b(\hat{k})(x_{00} + x_{10} + x_{20})) \tag{13}$$

$$\bar{w}x_{01}' = \frac{x_{00}x_{01}}{2} + \frac{x_{00}x_{11}}{4} + \frac{x_{00}x_{21}}{4} + \frac{x_{01}x_{00}}{2} + x_{01}x_{01} + \frac{x_{01}x_{10}}{4} + \frac{x_{01}x_{11}}{2} + \frac{x_{01}x_{20}}{4} + \frac{x_{01}x_{21}}{2} + \frac{1}{4}x_{11}x_{00}\left(1 - c_{sup}\right) + \frac{1}{2}x_{11}x_{01}\left(1 - c_{sup}\right) + \frac{1}{4}x_{21}x_{00}\left(1 - c_{sup}\right) + \frac{1}{2}x_{21}x_{01}\left(1 - c_{sup}\right) + \frac{1}{4}x_{10}x_{01}(1 - c_{sup})(1 + b(k)(x_{00} + x_{10} + x_{20})) + \frac{1}{4}x_{20}x_{01}(1 - c_{sup})(1 + b(\hat{k})(x_{00} + x_{10} + x_{20})) \tag{14}$$

$$\bar{w}x_{10}' = \frac{x_{00}x_{10}}{2} + \frac{x_{00}x_{11}}{4} + \frac{x_{01}x_{10}}{4} + \frac{1}{4}x_{11}x_{00}\left(1 - c_{sup}\right) + \frac{1}{2}x_{11}x_{10}\left(1 - c_{sup}\right) + \frac{1}{4}x_{11}x_{20}\left(1 - c_{sup}\right) + \frac{1}{4}x_{21}x_{10}(1 - c_{sup}) + \frac{1}{4}x_{10}x_{01}(1 - c_{sup})(1 + b(k)(x_{00} + x_{10} + x_{20})) + \frac{1}{2}x_{10}x_{11}(1 - c_{sup})(1 + b(k)(x_{00} + x_{10} + x_{20})) + \frac{1}{4}x_{10}x_{21}(1 - c_{sup})(1 + b(k)(x_{00} + x_{10} + x_{20})) + \frac{1}{2}x_{10}x_{00}(1 - c(k) + b(k)(x_{00} + x_{10} + x_{20})) + x_{10}x_{10}(1 - c(k) + b(k)(x_{00} + x_{10} + x_{20})) + \frac{1}{2}x_{10}x_{20}(1 - c(k) + b(k)(x_{00} + x_{10} + x_{20})) + \frac{1}{4}x_{20}x_{11}(1 - c_{sup})(1 + b(\hat{k})(x_{00} + x_{10} + x_{20})) + \frac{1}{2}x_{20}x_{10}(1 - c(\hat{k}) + b(\hat{k})(x_{00} + x_{10} + x_{20})) \tag{15}$$

$$\bar{w}x_{11}' = \frac{x_{00}x_{11}}{4} + \frac{x_{01}x_{10}}{4} + \frac{x_{01}x_{11}}{2} + \frac{1}{4}x_{11}x_{00}\left(1 - c_{sup}\right) + \frac{1}{2}x_{11}x_{01}\left(1 - c_{sup}\right) + \frac{1}{2}x_{11}x_{10}\left(1 - c_{sup}\right) + x_{11}x_{11}\left(1 - c_{sup}\right) + \frac{1}{4}x_{11}x_{20}\left(1 - c_{sup}\right) + \frac{1}{2}x_{11}x_{21}\left(1 - c_{sup}\right) + \frac{1}{4}x_{21}x_{10}\left(1 - c_{sup}\right) + \frac{1}{2}x_{21}x_{11}(1 - c_{sup}) + \frac{1}{4}x_{10}x_{01}(1 - c_{sup})(1 + b(k)(x_{00} + x_{10} + x_{20})) + \frac{1}{2}x_{10}x_{11}(1 - c_{sup})(1 + b(k)(x_{00} + x_{10} + x_{20})) + \frac{1}{4}x_{10}x_{21}(1 - c_{sup})(1 + b(k)(x_{00} + x_{10} + x_{20})) + \frac{1}{4}x_{20}x_{11}(1 - c_{sup})(1 + b(\hat{k})(x_{00} + x_{10} + x_{20})) \tag{16}$$

$$\bar{w}x_{20}' = \frac{x_{00}x_{20}}{2} + \frac{x_{00}x_{21}}{4} + \frac{x_{01}x_{20}}{4} + \frac{1}{4}x_{11}x_{20}\left(1 - c_{sup}\right) + \frac{1}{4}x_{21}x_{00}\left(1 - c_{sup}\right) + \frac{1}{4}x_{21}x_{10}\left(1 - c_{sup}\right) +$$

$\frac{1}{2}x_{21}x_{20}(1-c_{sup})+\frac{1}{4}x_{10}x_{21}(1-c_{sup})(1+b(k)(x_{00}+x_{10}+x_{20}))+\frac{1}{2}x_{10}x_{20}(1-$

$c(k)+b(k)(x_{00}+x_{10}+x_{20}))+\frac{1}{4}x_{20}x_{01}(1-c_{sup})(1+b(\hat{k})(x_{00}+x_{10}+x_{20}))+$

$\frac{1}{4}x_{20}x_{11}(1-c_{sup})(1+b(\hat{k})(x_{00}+x_{10}+x_{20}))+\frac{1}{2}x_5x_6(1-c_{sup})(1+b(\hat{k})(x_{00}+$

$x_{10}+x_{20}))+\frac{1}{2}x_{20}x_{00}(1-c(\hat{k})+b(\hat{k})(x_{00}+x_{10}+x_{20}))+\frac{1}{2}x_{20}x_{10}(1-c(\hat{k})+$

$b(\hat{k})(x_{00}+x_{10}+x_{20}))+x_{20}x_{20}(1-c(\hat{k})+b(\hat{k})(x_{00}+x_{10}+x_{20}))$ (17)

$\bar{w}x_{21}'=\frac{x_{00}x_{21}}{4}+\frac{x_{01}x_{20}}{4}+\frac{x_{01}x_{21}}{2}+\frac{1}{4}x_{11}x_{20}\left(1-c_{sup}\right)+\frac{1}{2}x_{11}x_{21}\left(1-c_{sup}\right)+\frac{1}{4}x_{21}x_{00}\left(1-c_{sup}\right)$

$+\frac{1}{2}x_{21}x_{01}\left(1-c_{sup}\right)+\frac{1}{4}x_{21}x_{10}\left(1-c_{sup}\right)+\frac{1}{2}x_{21}x_{11}\left(1-c_{sup}\right)+\frac{1}{2}x_{21}x_{20}\left(1-c_{sup}\right)$

$+x_{21}x_{21}(1-c_{sup})+\frac{1}{4}x_{10}x_{21}(1-c_{sup})(1+b(k)(x_{00}+x_{10}+x_{20}))+\frac{1}{4}x_{20}x_{01}(1-$

$c_{sup})(1+b(\hat{k})(x_{00}+x_{10}+x_{20}))+\frac{1}{4}x_{20}x_{11}(1-c_{sup})(1+b(\hat{k})(x_{00}+x_{10}+x_{20}))+$

$\frac{1}{2}x_{20}x_{21}(1-c_{sup})(1+b(\hat{k})(x_{00}+x_{10}+x_{20}))$. (18)

$\bar{w}$ is the average fitness of individuals in the current generation, and equals the sum of the right-hand side of the system of equations. The mutant trait distorter can invade when the equilibrium given by x_{00}*=0, x_{01}*=0, x_{10}*=1, x_{11}*=0, x_{20}*=0, x_{21}*=0 is unstable, which occurs when the leading eigenvalue of the Jacobian stability matrix for this equilibrium is greater than one. Testing for stability in this way, we find that the mutant trait distorter invades from rarity when $\Delta b>\Delta c$, where $\Delta b=b(\hat{k})-b(k)$, $\Delta c=c(\hat{k})-c(k)$.

The implication is that mutant trait distorters will invade if they approach a 'target' strength (k_{target}), corresponding to the level of trait distortion that would maximise the fitness of the gene[53], at which:

$$\frac{\partial b}{\partial k} = \frac{\partial c}{\partial k}. \tag{19}$$

In the absence of suppression, this target is the equilibrium level of distortion ($k^*=k_{target}$).

Equilibrium trait distorter and suppressor frequencies (long term evolution)

We ask what equilibrium state will arise after the invasion of a mutant trait distorter. We assume that the mutant trait distorter (D_2) is introduced from rarity when the resident trait distorter (D_1) has reached the population frequency given by q. We numerically iterate Supplementary Equations 13-18, over successive generations, until equilibrium has been reached. At equilibrium, for all parameter combinations (q,$t(k)$,$t(\hat{k})$,c_{sup},$c(k)$,$c(\hat{k})$), the resident trait distorter (D_1) is lost from the population (x_{10},x_{11}=0), with either the mutant trait distorter (D_2) and non-suppressor (S_0) at fixation (x_{20}^*=1), or the non-trait distorter at fixation alongside the suppressor at an internal equilibrium ($x_{00}^*+x_{01}^*=1$). The latter scenario arises if the mutant trait distorter triggers suppressor invasion ($c_{sup}(1+b(\hat{k})/2)<c(\hat{k})-b(\hat{k})/2$). This equilibrium arises because mutant trait distorter-presence gives the suppressor (S_1) a selective advantage, leading to high suppressor frequency, which in turn reverses the selective advantage of distortion, leading to trait distorter (D_1,D_2) loss and suppressor equilibration.

Given that mutant trait distorters will invade if they approach a ‘target’ strength (k_{target}), if the individual level cost associated with this target level of distortion ($c(k_{\text{target}})$) is sufficiently high relative to the cost of suppression (c_{sup}), so that the

following condition is satisfied, the equilibrium level of distortion will be $k^*=0$: $c_{sup}(1+b(k_{\text{target}})/2)<c(k_{\text{target}})-b(k_{\text{target}})/2$. If this condition is not satisfied the equilibrium level of distortion will be $k^*=k_{target}$ (Figure 3e).

Discussion

Although there have been no direct tests, our predictions are consistent with data on imprinted genes. There is no evidence that traits influenced by imprinted genes deviate significantly from individual level optima under normal development[52]. Significant deviation is only observed when imprinted genes are deleted, implying that imprinted trait distorters are either suppressed, or counterbalanced by oppositely imprinted genes pulling the trait in the opposite direction[63,76]. Furthermore, although many different parties (coreplicons) have vested interests in genomic imprinting, our analysis suggests why the unimprinted majority could win control[77]. This could help explain both why imprinting appears to be relatively rare within the genome[50,54,66], and why imprints are removed and re-added every generation in mice, handing control of genomic patterns of imprinting to unimprinted genes[54,77,78].

Supplementary Note 5

Horizontal Gene Transfer and Public Goods

Bacteria produce and excrete many extracellular factors that provide a benefit to the local population of cells and so can be thought of as public goods[79]. We modelled the evolution of investment in a public good in a large, clonally reproducing population. We assume a public good that costs c to produce, and provides a benefit b to the group. We assume a well-mixed population, meaning genetic relatedness at vertically inherited genes is zero ($R_{vertical}=0$), and so indirect fitness benefits cannot favour public good production at the individual level ($R_{vertical}b=0<c$)[51,74,75,80,81]. There are also direct fitness benefits of public good production, which arise because producers of public goods receive a fraction of the benefit (b) they confer on the group, but we assume that the population is sufficiently large and well mixed that direct fitness benefits cannot favour public good production at the individual level. This means that public good production is disfavoured at the individual level.

We consider a selfish genetic element that resides on a mobile locus (horizontal & vertical transmission) and may gain a propagation advantage by upregulating individual public goods investment[82-86]. The genes that do not gain a propagation advantage from increased public goods production comprise the non-mobile loci (vertical transmission). Non-mobile loci comprise most of the genome, and so constitute the majority within the parliament of genes. We focus our analyses on when a mobile trait distorter and a non-mobile suppressor can spread. The purpose of this model is to illustrate how selection will act on selfish mobile genes and their suppressors.

Model assumptions

We consider a public goods gene (D_1) that competes against a non-trait distorter (D_0) at a mobile locus. The trait distorter (D_1) increases public goods investment by some amount (k), at a fitness cost to the individual ($c(k)$) and benefit shared within the group ($b(k)>c(k)$) that are both monotonically increasing functions of investment $\left(\frac{\partial\{b,c\}}{\partial k} \geq 0\right)$.

We assume the following lifecycle. Individuals in a large, effectively infinite, population randomly aggregate into smaller social groups (*patches*). Individuals then randomly pair up within their patch, and horizontal gene transfer occurs, with certainty, within pairs that are genetically dissimilar at the mobile locus[87,88]. Alternative assumptions about the probability of horizontal gene transfer do not change our qualitative results (Scott, unpublished). Only one allele at the mobile locus is transferrable in each patch, and each allele at the mobile locus is transferrable in an equal proportion of patches. We denote those patches in which the non-trait distorter (D_0) is transferred as "type 1" patches, and those patches in which the trait distorter (D_1) is transferred as "type 2" patches. Individuals may then produce public goods, which are shared within patches, before the population re-merges, and individuals reproduce in proportion to their fitness before dying (non-overlapping generations), with progeny inheriting all alleles from their parent (perfect inheritance).

Trait Distorter Spread

We respectively take $\bar{p}$ and $\bar{p}''$ as the population frequency of the trait distorter (D_1) at the start of two consecutive generations, and p'_j as the average frequency of the trait distorter (D_1) in patches of type j after horizontal gene transfer ($j\in\{1,2\}$), with $p'_{j=1}$ = $\bar{p}$ + $\bar{p}$(1-$\bar{p}$) and $p'_{j=2}$ = $\bar{p}$ – $\bar{p}$(1-$\bar{p}$). The population frequency of the trait distorter in the latter generation ($\bar{p}''$) is:

$$\bar{p}'' = \frac{p'_{j=1}\left(1+p'_{j=1}b(k)-c(k)\right)+p'_{j=2}\left(1+p'_{j=2}b(k)-c(k)\right)}{2+(b(k)-c(k))\left(p'_{j=1}+p'_{j=2}\right)}, \tag{20}$$

where the denominator denotes average individual fitness. Stable equilibria occur for $\bar{p}$=$\bar{p}''$=p^* and $\left.\frac{\partial \bar{p}''}{\partial \bar{p}}\right|_{\bar{p}=p^*} < 1$, which occurs when $p^* = \left\{0, \left(1+\sqrt{1-\frac{4c(k)}{b(k)}}\right)/2\right\}$.

Unstable equilibria occur for $\bar{p}$=$\bar{p}''$=$\bar{p}^*$ and $\left.\frac{\partial \bar{p}''}{\partial \bar{p}}\right|_{\bar{p}=p^*} > 1$, which occurs when $p^* = \left\{\left(1-\sqrt{1-\frac{4c(k)}{b(k)}}\right)/2, 1\right\}$. Therefore, the trait distorter (D_1) exhibits positive and negative frequency dependence, meaning it can only invade if introduced at high enough frequency $\left(\bar{p} > \left(1-\sqrt{1-\frac{4c(k)}{b(k)}}\right)/2\right)$, reaching a polymorphism below fixation $\left(p^* = \left(1+\sqrt{1-\frac{4c(k)}{b(k)}}\right)/2\right)$ (Supplementary Figure 10). Frequency dependence arises because, when there is low genetic diversity at the mobile locus (p→0/1), there is less generational horizontal gene transfer, and correspondingly lower patch relatedness, which dissipates the trait distorter's selective advantage[82]. A trait distorter (D_1) is more likely to invade and reach a high population frequency if it produces a public good associated with a large benefit to cost ratio (b/c)[82-84,89-91].

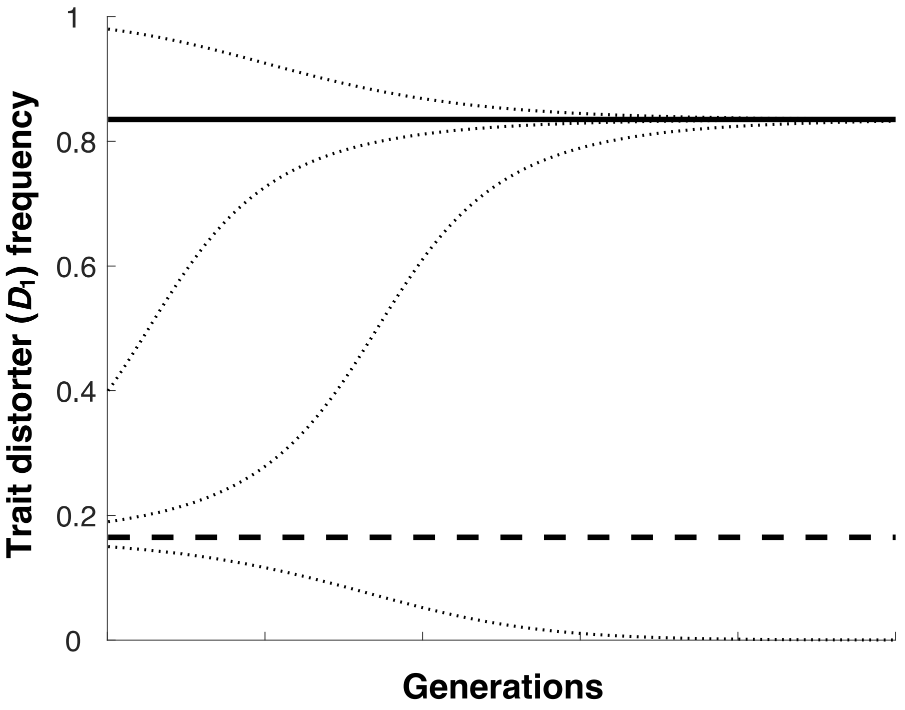

Supplementary Figure 10. Spread of the trait distorter (D_1) in the absence of suppression. The trait distorter (D_1) is introduced at some frequency, and equilibrates over successive generations (dotted lines indicate trajectories corresponding to different initial trait distorter frequencies). The solid and dashed lines are, respectively, the stable $\left(p^* = \left(1 + \sqrt{1 - \frac{4c(k)}{b(k)}}\right)/2\right)$ and unstable $\left(p^* = \left(1 - \sqrt{1 - \frac{4c(k)}{b(k)}}\right)/2\right)$ equilibria. If introduced at a frequency greater than the unstable internal equilibrium, trait distorter (D_1) frequency reaches the stable internal equilibrium, without going to fixation (negative frequency dependence). If introduced at a frequency lower than the unstable internal equilibrium, trait distorter (D_1) frequency goes to zero (positive frequency dependence).

Spread of a suppressor and consequences for the organism

We consider a suppressor allele (S_1) that competes against a non-suppressor (S_0) at a non-mobile locus. Suppressors of mobile elements are widespread and may silence elements before they are translated, through gene methylation and RNAi[92]. We respectively take $\bar{x}_i$ and $\bar{x}_i$" as the population genotype frequencies at the start of two consecutive generations, with the subscript $i \in \{00,01,10,11\}$ denoting the

respective genotypes: $\{D_0/S_0, D_0/S_1, D_1/S_0, D_1/S_1\}$. We take x_{ij}' as the average frequency of genotype i in patches of type j after horizontal gene transfer ($j \in \{1,2\}$), with $x_{001}' = \bar{x}_{i=00} + (\bar{x}_{i=00} + \bar{x}_{i=01})\bar{x}_{i=10}$, $x_{011}' = \bar{x}_{i=01} + (\bar{x}_{i=00} + \bar{x}_{i=01})\bar{x}_{i=11}$, $x_{101}' = \bar{x}_{i=10} - (\bar{x}_{i=00} + \bar{x}_{i=01})\bar{x}_{i=10}$, $x_{111}' = \bar{x}_{i=11} - (\bar{x}_{i=00} + \bar{x}_{i=01})\bar{x}_{i=11}$, $x_{002}' = \bar{x}_{i=00} - (\bar{x}_{i=10} + \bar{x}_{i=11})\bar{x}_{i=1}$, $x_{012}' = \bar{x}_{i=01} - (\bar{x}_{i=10} + \bar{x}_{i=11})\bar{x}_{i=01}$, $x_{102}' = \bar{x}_{i=10} + (\bar{x}_{i=10} + \bar{x}_{i=11})\bar{x}_{i=00}$, $x_{112}' = \bar{x}_{i=11} + (\bar{x}_{i=10} + \bar{x}_{i=11})\bar{x}_{i=01}$. The population genotype frequencies in the latter generation ($\bar{x}_i''$) are:

$$W\bar{x}_{00}'' = \sum_{j=1}^{j=2}(x_{00j}'(1 + x_{10j}'b(k))), \quad (21)$$

$$W\bar{x}_{01}'' = \sum_{j=1}^{j=2}(x_{01j}'(1 + x_{10j}'b(k))), \quad (22)$$

$$W\bar{x}_{10}'' = \sum_{j=1}^{j=2}(x_{10j}'(1 + x_{10j}'b(k) - c(k))), \quad (23)$$

$$W\bar{x}_{11}'' = \sum_{j=1}^{j=2}(x_{11j}'(1 + x_{10j}'b(k))(1 - c_{sup}))), \quad (24)$$

where W is average individual fitness, equal to the sum of the right-hand sides of the system of equations.

We numerically iterated these recursions, for a range of parameter values (b,c,c_{sup}), and for different initial frequencies of the trait distorter (D_1) to find the trait distorter (D_1) and suppressor (S_1) frequencies at equilibrium, and the resulting average trait distortion ($x_{10}\,k$). We found that, when distortion is weak (low k), suppressors are not favoured, but the trait distorter has relatively little impact at the individual level. For example, when the cost of suppression is c_{sup}=0.05, and the cost and benefit of public goods production are $c_{HGT}=k$ (linear cost) and $b_{HGT}=8k^{0.9}$ (relatively large,

decelerating benefit), unsuppressed trait distorters cannot upregulate public goods by more than k=c_{HGT}=0.08 (Figure 3c).

We found that the suppressor invades from rarity, in response to a trait distorter at equilibrium $\left(x_{10}^{*}=\left(1+\sqrt{1-\frac{4c(k)}{b(k)}}\right)/2\right)$, above a threshold level of distortion. If the suppressor invades, it increases in frequency until the trait distorter's (D_1) selective advantage is reversed and the trait distorter is lost from the population; the suppressor (S_1) then equilibrates (Figure 3c). A trait distorter (D_1) is more likely to evade suppression if it produces a public good associated with a large benefit to cost ratio ($b(k)/c(k)$) and if there is a high cost of suppression (c_{sup})[93].

Evolution of trait distortion

We ask when a mutant trait distorter (D_2) of strength ($\hat{k}$) will invade against a resident trait distorter (D_1) that is unsuppressed and at equilibrium ($\hat{k}\neq k$). We denote those patches in which the mutant trait distorter (D_2) is transferred as "type 3" patches. We use the subscript $i\in\{00,01,10,11,20,21\}$ to denote the respective genotypes $\{D_0/S_0,D_0/S_1,D_1/S_0,D_1/S_1,D_2/S_0,D_2/S_1\}$, and $j\in\{1,2,3\}$ to denote patch type. Average genotype frequencies in each patch type after horizontal gene transfer (x_{ij}') are given by: $x_{001}'=\bar{x}_{i=00}+(\bar{x}_{i=00}+\bar{x}_{i=01})(\bar{x}_{i=10}+\bar{x}_{i=20})$, $x_{011}'=\bar{x}_{i=01}+(\bar{x}_{i=00}+\bar{x}_{i=01})(\bar{x}_{i=11}+\bar{x}_{i=21})$, $x_{101}'=\bar{x}_{i=10}-(\bar{x}_{i=00}+\bar{x}_{i=01})\bar{x}_{i=10}$, $x_{111}'=\bar{x}_{i=11}-(\bar{x}_{i=00}+\bar{x}_{i=01})\bar{x}_{i=11}$, $x_{201}'=\bar{x}_{i=20}-(\bar{x}_{i=00}+\bar{x}_{i=01})\bar{x}_{i=20}$, $x_{211}'=\bar{x}_{i=21}-(\bar{x}_{i=00}+\bar{x}_{i=01})\bar{x}_{i=21}$, $x_{002}'=\bar{x}_{i=00}-(\bar{x}_{i=10}+\bar{x}_{i=11})\bar{x}_{i=00}$, $x_{012}'=\bar{x}_{i=01}-(\bar{x}_{i=10}+\bar{x}_{i=11})\bar{x}_{i=01}$, $x_{102}'=\bar{x}_{i=10}+(\bar{x}_{i=10}+\bar{x}_{i=11})(\bar{x}_{i=00}+\bar{x}_{i=20})$, $x_{112}'=\bar{x}_{i=11}+(\bar{x}_{i=10}+\bar{x}_{i=11})(\bar{x}_{i=01}+\bar{x}_{i=21})$, $x_{202}'=\bar{x}_{i=20}-(\bar{x}_{i=10}+\bar{x}_{i=11})\bar{x}_{i=20}$, $x_{212}'=\bar{x}_{i=21}-(\bar{x}_{i=10}+\bar{x}_{i=11})\bar{x}_{i=21}$, $x_{003}'=$

$\bar{x}_{i=00} - (\bar{x}_{i=20} + \bar{x}_{i=21})\bar{x}_{i=00}$, $x_{013}' = \bar{x}_{i=01} - (\bar{x}_{i=20} + \bar{x}_{i=21})\bar{x}_{i=01}$, $x_{103}' = \bar{x}_{i=10} - (\bar{x}_{i=20} + \bar{x}_{i=21})\bar{x}_{i=10}$, $x_{113}' = \bar{x}_{i=11} - (\bar{x}_{i=20} + \bar{x}_{i=21})\bar{x}_{i=11}$, $x_{201}' = \bar{x}_{i=20} + (\bar{x}_{i=20} + \bar{x}_{i=21})(\bar{x}_{i=00} + \bar{x}_{i=10})$, $x_{211}' = \bar{x}_{i=21} + (\bar{x}_{i=20} + \bar{x}_{i=21})(\bar{x}_{i=01} + \bar{x}_{i=11})$. We write recursions detailing the generational genotype frequency changes:

$$W\bar{x}_{00}'' = \sum_{j=1}^{j=3}(x_{00j}'(1 + x_{10j}'b(k) + x_{20j}'b(\hat{k}))), \quad (25)$$

$$W\bar{x}_{01}'' = \sum_{j=1}^{j=3}(x_{01j}'(1 + x_{10j}'b(k) + x_{20j}'b(\hat{k}))), \quad (26)$$

$$W\bar{x}_{10}'' = \sum_{j=1}^{j=3}(x_{10j}'(1 + x_{10j}'b(k) + x_{20j}'b(\hat{k}) - c(k))), \quad (27)$$

$$W\bar{x}_{11}'' = \sum_{j=1}^{j=3}(x_{11j}'(1 + x_{10j}'b(k) + x_{20j}'b(\hat{k}))(1 - c_{sup}))), \quad (28)$$

$$W\bar{x}_{20}'' = \sum_{j=1}^{j=3}(x_{20j}'(1 + x_{10j}'b(k) + x_{20j}'b(\hat{k}) - c(\hat{k}))), \quad (29)$$

$$W\bar{x}_{21}'' = \sum_{j=1}^{j=3}(x_{21j}'(1 + x_{10j}'b(k) + x_{20j}'b(\hat{k}))(1 - c_{sup}))), \quad (30)$$

where *W* is average individual fitness, equal to the sum of the right-hand sides of the system of equations.

We assume that trait distorter strength (k) is initially low, and introduce successive mutant trait distorters (D_2), each deviating only slightly from the trait distorters from which they are derived, until one fails to displace the resident trait distorter. The strength of the non-invadable allele gives the equilibrium level of distortion under δ-weak selection[49]. We find that, if the rate of decrease in marginal cooperative benefits $\left(-\frac{d^2b}{dk^2}\right)$ is high relative to the rate of increase in marginal cooperative costs $\left(\frac{d^2c}{dk^2}\right)$, distortion ($k^*$) evolves to be low, and the suppressor (S_1) may not invade. Otherwise, stronger trait distorters (D_2) successively invade, bringing trait distorter

strength above the threshold level at which the suppressor (S_1) spreads, with the end result that trait distorters are suppressed and lost from the population, with no trait distortion at equilibrium (k^*=0) (Figure 3f).

Discussion

We lack empirical data that would allow us to test our model of mobile public goods genes. Genes associated with extracellular traits, which could represent cooperative public goods, appear to be overrepresented on mobile elements[91]. However, this may be nothing to do with cooperation *per se* – genes involved with adaptation to new environments might be more likely to be horizontally acquired, and extracellular traits might be especially important in adaptation to new environments[84-87,94].

Supplementary Note 6

Suppressor Conditionality

We assumed in our Equilibrium and Dynamics models (Main Text) that suppressors are only expressed in the presence of their target trait distorters (facultative). We generalise our Equilibrium models (Main Text) by defining the parameter ψ as the "conditionality" of the suppressor ($0 \leq \psi \leq 1$). For full conditionality (ψ=1), the suppressor is facultative. For zero conditionality (ψ=0), the suppressor is obligate, meaning it is fully expressed when the trait distorter is absent. For intermediate conditionality ($0<\psi<1$), the suppressor is partially expressed when the trait distorter is absent. As a result, the suppressor incurs a cost of c_{sup} on the individual when the trait distorter is present, and a cost of $(1-\psi)*c_{sup}$ when the trait distorter is absent.

In the facultative suppressor case (ψ=1), considered in the main text, the fitness of D_0/S_0 D_0/S_1 and D_0/S_1 D_0/S_1 individuals, which have a suppressor but not a trait distorter, is 1. Now, in the generalised scenario, the fitness of these individuals is:

$$1-(1-\psi)*c_{sup}. \tag{31}$$

Amending Equations 2-5 & 8-13 (main text) according to this small change, and repeating the analysis described in the Methods section (main text), reveals that the suppressor invasion condition ($c_{sup}<c_{trait}(k)$) and the stronger-trait distorter invasion condition ($\Delta t(1-c_{trait}(\hat{k}))>\Delta c_{trait}$) are unchanged. The suppressor invasion condition is unchanged because an invading suppressor can only gain a selective advantage if it

finds itself in the same individual as a trait distorter, and in such a scenario, it will confer the full cost of c_{sup} regardless of its conditionality (ψ). The stronger-trait distorter invasion condition is unchanged because it is derived for an equilibrium in which the suppressor is absent.

However, suppressor conditionality affects trait distorter-suppressor dynamics in a subtle way. In the facultative suppressor case (ψ=1), considered in the main text, the spread of the suppressor from rarity causes the trait distorter to lose its selective advantage and be eliminated from the population, leading to an absence of distortion at the individual level. This occurs because, under suppression, the trait distorter (D_1) gains no transmission advantage over the non-trait distorter (D_0), but is associated with a cost of c_{sup} arising from facultative suppressor expression. The non-trait distorter (D_0) does not pay this cost, so gains a selective advantage under suppression, and spreads at the expense of the trait distorter (D_1).

For suppressors with intermediate conditionality ($0<\psi<1$), suppressor spread means that the trait distorter (D_1) pays a cost of c_{sup} and the non-trait distorter (D_0) pays a smaller cost of $((1-\psi)*c_{sup})$. As a result, as the suppressor spreads to high population frequency, the non-trait distorter (D_0) gains a selective advantage, and spreads at the expense of the trait distorter (D_1). However, the selective advantage of the non-trait distorter (D_0), over the trait distorter (D_1), under suppression, is weaker when the suppressor has intermediate conditionality ($0<\psi<1$), compared to when it is fully facultative (ψ=1). As a result, the time taken for the trait distorter (D_1) to fall to

negligible population frequency is increased if suppressors are not fully facultative ($0<\psi<1$).

For obligate suppressors ($\psi=0$), the trait distorter (D_1) has equal fitness to the non-trait distorter (D_0) under suppression, as both face the full cost of c_{sup}, owing to obligate suppressor expression. This means that trait distorters are not purged after suppression, and though the trait is fully restored to optimality as a result of suppressor fixation, individuals continue to pay the cost of c_{sup} at equilibrium. The residual cost (c_{sup}) is an artefact of the conflict, and will remain, to the detriment of population (absolute) mean fitness, until a conditional ($\psi>0$) suppressor arises by mutation and selectively displaces the obligate one.

Supplementary Note 7

Dynamics Models (Additional Figures)

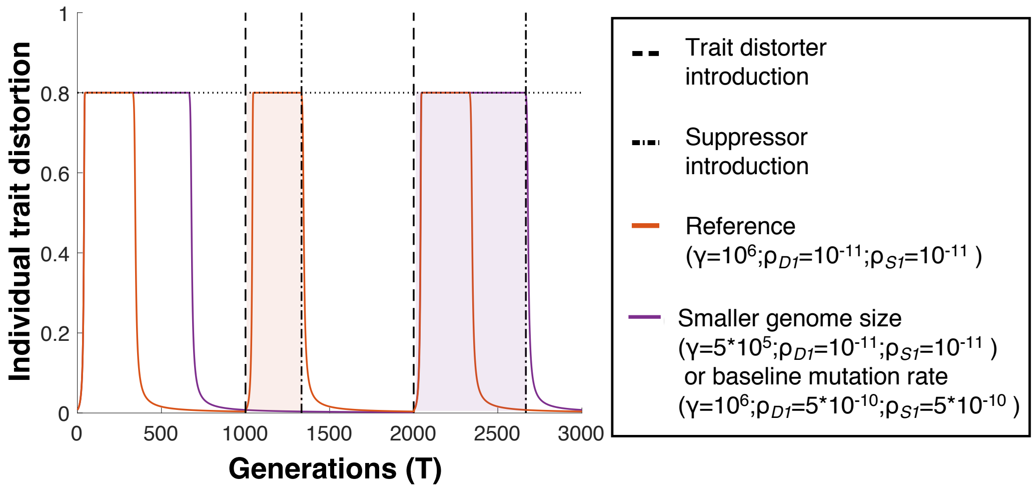

Supplementary Figure 11. The effect of genome size (γ) / mutation rate (ρ) when trait distorters do not co-segregate. Individual trait distortion is plotted over evolutionary time. We introduced trait distorters (D_1) deterministically at new loci every $1/(\theta\gamma\rho_{D1})$ generations, and their dedicated suppressors after a lag of $1/((1-\theta)\gamma\rho_{sup})$ generations. Individual trait distortion increases and decreases cyclically over evolutionary time, between peaks of k and troughs of 0.

Individual trait distortion is plotted for three different parameter regimes. The first parameter regime is plotted as a reference, and represented by the red line ($\gamma=10^6$; $\rho_{D1}=10^{-11}$,$\rho_{S1}=10^{-11}$). The second parameter regime has a half-sized genome size relative to the reference, with an unchanged baseline mutation rate ($\gamma=5*10^5$; $\rho_{D1}=10^{-11}$,$\rho_{S1}=10^{-11}$). The third parameter regime has a half-sized baseline mutation rate relative to reference, with an unchanged genome size ($\gamma=10^6$; $\rho_{D1}=5*10^{-10}$,$\rho_{S1}=5*10^{-10}$). Proportional changes in genome size (γ) have identical effects to proportional changes in baseline mutation rate (ρ), and therefore, the second and third parameter regimes lead to the same outcome, which is represented by the purple line.

Owing to rapid gene frequency equilibration after trait distorter / suppressor introduction, the periodic functions (red and purple lines) can be approximated as rectangles. A decrease in genome size (γ) or baseline mutation rate (ρ) leads to an increase in the width, and therefore area (shaded regions), of the rectangles, but a corresponding decrease in the density of the rectangles. Therefore,

the average trait distortion across evolutionary time, which is given by the area under the curve, and approximated by $\frac{k\theta\rho_{D_1}}{(1\text{-}\theta)\rho_{S_1}}$ (Equation 6), is unaffected by genome size (γ) and baseline mutation rate (ρ). These numerical solutions assume the following parameter values: c_{sup}=0.1; t=k, c_{trait}=$k/2$, k=0.8.

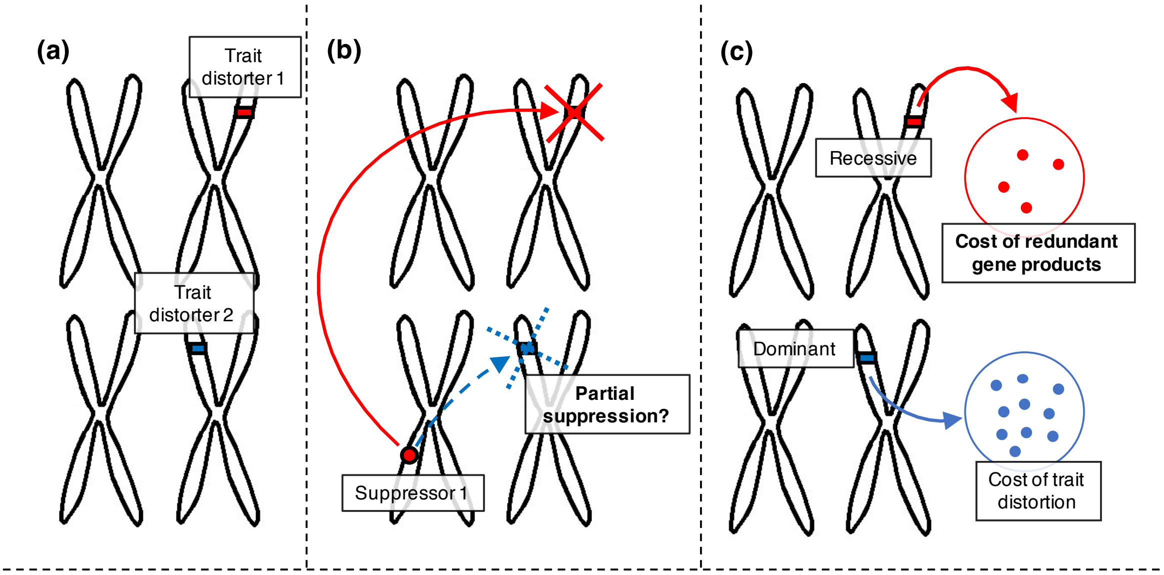

(d)

Trait distorter sophistication	Rate of introduction	Partial suppression of non-targets?	Cost of redundant gene products?
Low (D_{1L})	Fast	✔	Large
High (D_{1H})	Slow	✘	Small

Supplementary Figure 12. Types of trait distorter interaction. (a) Trait distorters may arise at different loci within the cabal (e.g. red and blue rectangle markers). Low-sophistication trait distorters are more mutationally accessible than high-sophistication trait distorters (ρ_{D1L}=2*ρ_{D1H}), so arise more frequently.

(b) A dedicated suppressor of a trait distorter at a specific locus (e.g. red rectangle marker) may arise at some locus within the commonwealth (e.g. red circle marker). The dedicated suppressor suppresses its target trait distorter with full strength (red solid arrow). If trait distorters are low-sophistication, dedicated suppressors also suppress non-target trait distorters (e.g. blue rectangle marker) with partial (z=0.5) strength (blue dashed arrow). High-sophistication trait distorters are invulnerable to non-target suppression (z=0).

(c) Of all trait distorters across a genome, the one that is most trait-distorting after suppression exhibits inter-locus dominance, and distorts the individual trait. Expression of the inter-locus recessive trait distorters results in an individual-level cost (c_{rec}), which is greater for low-sophistication trait distorters $\left(c_{trait}(Dist) = \frac{c_{rec}(Waste)}{|I_{distorter}|-1} \geq 0\right)$ than high-sophistication ones $\left(c_{trait}(Dist) = \frac{5(c_{rec}(Waste))}{3(|I_{distorter}|-1)} \geq 0\right)$.

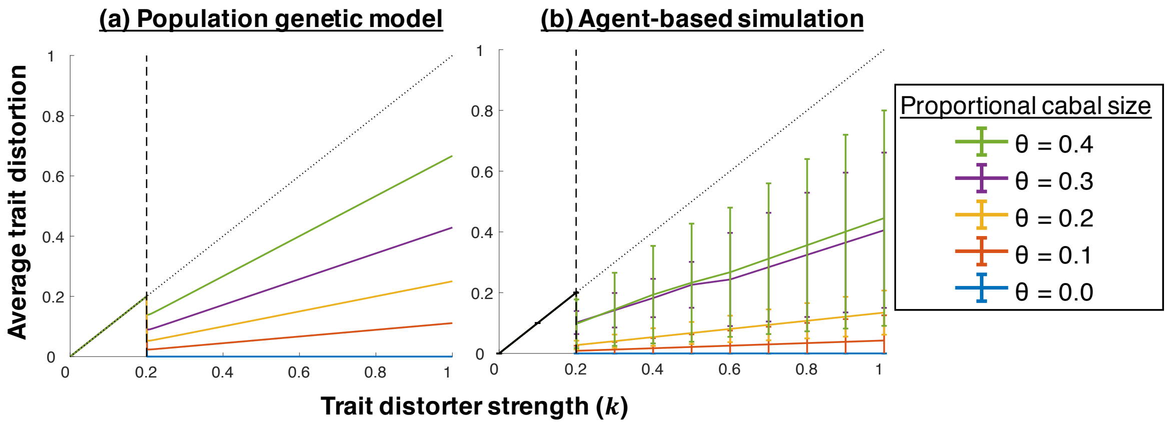

Supplementary Figure 13. Comparison of population genetic and agent-based simulation results when trait distorters do not co-segregate. Trait distorters (D_1) and their dedicated suppressors (S_1) are continuously introduced, from rarity, at different loci within the cabal and the commonwealth respectively. The resulting average individual trait distortion, taken over evolutionary time, is plotted, for different proportional cabal sizes (θ), against the extent to which the trait distorters cause trait values to deviate from the individual optimum (k). The following parameter values are assumed: c_{sup}=0.1; t=k, c_{trait}=$k/2$, γ=10^6, ρ_{S1}=10^{-11},ρ_{D1}=10^{-11}. On these assumptions, trait distorters favouring suppression (c_{sup}<$c_{trait}(k)$), which lie to the right of the dashed lines, scarcely co-segregate.

Part (a) plots numerical solutions for the population genetic model described in *Methods: Long term trait distortion (exact numerical solution)*. Part (b) plots the average results from 4 runs of the agent-based simulation model described in *Methods: Agent-based simulation (multiple loci; discrete)*, where each simulation is run for T_{end}=10^6 generations, and where error bars represent one standard deviation in each direction. For the simulations, we arbitrarily assume that trait distorters are low-sophistication (D_{1L}) as opposed to high-sophistication (D_{1H}). However, this choice is inconsequential given that the characteristics of trait distorter interaction do not affect average trait distortion when trait distorters scarcely co-segregate.

Given the exceedingly low probabilities of trait distorter / suppressor introduction in these parameterisations (very high stochasticity), the simulation results underestimate average trait distortion, and are highly variable (large error bars). The simulation results are underestimates because, as individual simulation runs are finite (T_{end}), they may end before rare trait distorters have been completely purged. Nevertheless, the results of the two models are consistent, and increasingly converge as simulation run times (T_{end}) are increased.

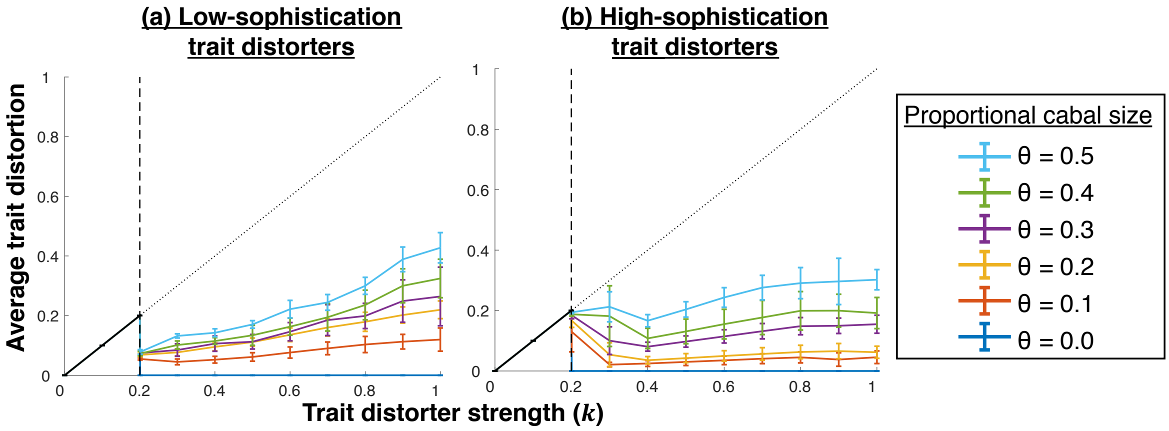

Supplementary Figure 14. The effect of trait distorter strength (k) when trait distorters may co-segregate. Trait distorters (D_1) and their dedicated suppressors (S_1) are continuously introduced, from rarity, at different loci within the cabal and the commonwealth respectively (*Methods: Agent-based simulation (multiple loci; discrete)*). The resulting average individual trait distortion, taken over T_{end}=30,000 generations, is plotted as an average over 4 simulation runs, with error bars representing one standard deviation in each direction. Average trait distortion is plotted for different levels of trait distorter sophistication (low; high), and different proportional cabal sizes (θ), against the extent to which the trait distorters cause trait values to deviate from the individual optimum (k).

Weaker trait distorters (k) are suppressed and purged more slowly than stronger ones, and are therefore more likely to co-segregate. As a result, when trait distorters are (a) low-sophistication (D_{1L}), average trait distortion is pulled below linearity (Equation 6; Main Text) when trait distorters are weak, leading to an accelerating relationship between average trait distortion and trait distorter strength (k). When trait distorters are (b) high-sophistication (D_{1H}), average trait distortion is pulled above linearity (Equation 6; Main Text) when trait distorters are weak, meaning the difference in average trait distortion caused by trait distorters of different strengths (k) is reduced (flatter relationship).

For reduced proportional cabal size (θ), average trait distortion is reduced, and the relationship between trait distorter strength (k) and average trait distortion tends towards linearity (Equation 6; Main Text).

The following parameter values were taken: c_{sup}=0.1, t=k, c_{trait}=$Dist/2$, $\gamma=10^6$, $\rho_{S1}=4*10^{-9}$, $\rho_{D1L}=4*10^{-9}$, $\rho_{D1H}=2*10^{-9}$. For these parameters, genome size (γ) and baseline mutation rate

(ρ_{S1}/ρ_{D1L}/ρ_{D1H}) are not exceedingly high. As a result, the increased trait distortion achieved by high-sophistication trait distorters as a result of productive interaction whilst co-segregating is roughly offset by the increased trait distortion achieved by low-sophistication trait distorters as a result of higher mutational accessibility. This is why trait distorter sophistication has a relatively small effect on average trait distortion, as can be seen by comparing (a) and (b).

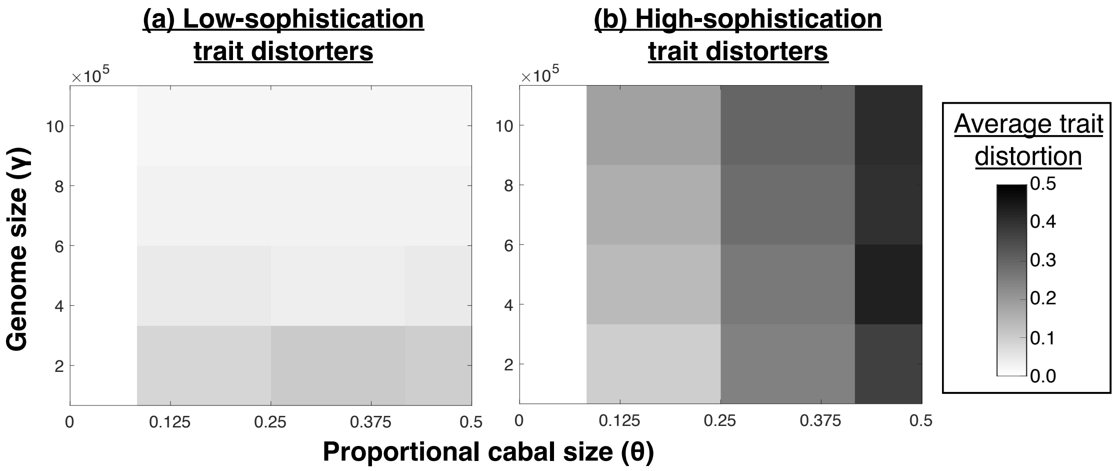

Supplementary Figure 15. Evolution of trait distortion and suppression across the genome. The figures show how the average level of trait distortion depends upon the cabal (θ) and genome (γ) size, for both (a) low- and (b) high-sophistication trait distorters. The results are from our agent-based simulation (*Methods: Agent-based simulation (multiple loci; continuous)*), where trait distorters (D_1) and their dedicated suppressors (S_1) are continuously introduced. Trait distorters and suppressors vary continuously in strength, and are free to evolve. Each block is the average of 30 simulation runs, each over T_{end}=30,000 generations. Average trait distortion increases with cabal size (θ). Low-sophistication trait distorters interact counter-productively whilst co-segregating, and so average trait distortion decreases with genome size (γ). High-sophistication trait distorters interact productively whilst co-segregating, and so average trait distortion increases with genome size (c_{sup}=0.01, *t*=*k*, c_{trait}=*Dist/2,* $\rho_{S1}=4*10^{-9}$, $\rho_{D1L}=4*10^{-9}$, $\rho_{D1H}=2*10^{-9}$).

Supplementary Note 8

Real-World Estimates of Proportional Cabal Size

Y chromosome cabal in *Drosophila melanogaster*

- A review of *Drosophila* Y chromosome evolution pulls together indirect evidence to suggest that, although only 12 genes are currently known, there is an upper bound of 20 genes on the *D.melanogaster* Y chromosome[105]. We use this upper bound for our calculation of proportional cabal size, meaning proportional cabal size is likely to be an overestimate.
- The total number of genes in the *D.melanogaster* genome is 17,684,[106] meaning the proportional cabal size can be calculated as θ = 20/17684 = 0.001 (1*sf*).

Cytoplasmic element & X chromosome cabal in humans

- In humans, the only cytoplasmic elements that carry transcribed genes are the mitochondria. Human mitochondria bear 37 genes[107].
- The number of genes on the human X chromosome (protein coding genes plus non-coding RNA genes) is 1515 (Ensembl release 97 - July 2019)[108].
- The total number of genes in the human genome is 42611[109].
- In human females, the proportional size of the cabal favouring female sex ratio distortion can be calculated as θ = (37 + 1515) / 42611 = 0.04 (*1sf*).

Plasmid cabal in *Escherichia coli*

- Different *E.coli* individuals will carry different numbers and types of plasmids. We therefore draw a random sample of 139 *E.coli* strains from the 875 *E.coli*

strains for which complete genome sequences are publicly available (Genbank Refseq; ftp://ftp.ncbi.nih.gov/genomes).

- For each strain in our sample, we calculate proportional cabal size by counting the number of genes that are on plasmids, and dividing this by the total number of genes in the individual.
- Averaging over strains, we calculate proportional cabal size as $\theta=0.036$ (2 *sf*).

Supplementary Discussion 1

Relation to Gardner & Úbeda (2017) and Grafen's Formal Darwinism

Gardner & Welch (2011)[95] provided formal justification for the view that genes evolve so as to maximise their own fitness (*selfish gene theory*). Specifically, they showed that, over evolutionary time, the allele-variants that come to occupy positions at loci are those variants that maximise the inclusive fitness of the gene. Following this formalism, Gardner & Úbeda (2017)[53] defined intra-genomic conflict as instances where the evolutionary interests of different genes, as determined by what maximises their respective inclusive finesses, do not coincide. The approach of Gardner & Úbeda (2017)[53] is useful because it clarifies the evolutionary "battleground" over which intra-genomic conflict can play out. If a "battleground" model establishes the *causes* of intra-genomic conflict, then a "resolution" model addresses the *consequences*. Our models are resolution models, and complementary to the battleground models described in Gardner & Úbeda (2017)[53].

The theoretical justification for organismal fitness maximisation is found the optimisation models of Grafen's Formal Darwinism project[96]. This formalism assumes that organism phenotype is controlled solely by genes in a single co-replicon, in which genes are unimprinted, autosomal and inherited in Mendelian fashion[6]. Our models follow Burt & Trivers (2006)[52] in taking organismal fitness maximisation as a starting point, and then addressing how robust this formalism is once nascent selfish genetic elements, residing in minority co-replicons, can gain

some control over organism phenotype. Our models are therefore complementary to those of Grafen's Formal Darwinism project.

Individual fitness maximisation: emergence versus maintenance

Specifically, our models show that, if individuals are maximising their fitness with respect to a given trait under potential conflict, then attempts to distort the trait from individual fitness maximisation, driven by selfish genetic elements arising in coreplicons representing minority-interests in the genome (cabal), will by and large be futile, unless the cabal is relatively large in size (approaching half of the genome). Therefore, our models provide justification for the idea that, once an organism has obtained fitness maximisation, it cannot, in general, be appreciably distorted by the subsequent invasion of trait-distorting elements.

However, there is a bias in our methodology. We assumed that the organism is already maximising its fitness, and then showed that subsequent distortions from this maximand will often be negligible. This bias is evident in the strategy set afforded to different alleles across the genome: the minority-interest within the genome (cabal) can only exert influence over the trait via 'trait distorters' (they distort the trait– they cause a shift away from the norm), whereas the majority-interest within the genome (commonwealth) can only exert influence over the trait via 'suppressors' (they restore – they cause a shift back towards the norm). Therefore, what our models really show is that, for traits under intragenomic conflict, individual fitness maximisation can be maintained. They do not show that individual fitness maximisation is obtained in the first place (emerges). The question of whether

individual fitness maximisation emerges when traits are underpinned by conflicting coreplicons is a direction for future research.

Supplementary Discussion 2

Simple Selfish Genetic Elements vs Trait Distorters

We draw a distinction between two types of selfish genetic element. “Simple” selfish genetic elements (SGEs), such as transposons and simple meiotic drivers, do not need to manipulate organism traits in order to give themselves a selfish propagation advantage. The spread of a simple SGE may have detrimental consequences for the organism, for example due to the disruptive act of driving itself[97]. However, these costs are disfavoured across the whole genome, including by the simple SGE in question, and there will be unanimous selection across the genome to attenuate these costs. The spread of simple SGEs therefore does not generate intra-genomic conflict over organism form. Their existence therefore does not compromise organismal design (individual fitness maximisation).

Simple meiotic drivers may incur fertility costs from the act of driving, which will be alleviated once the driver reaches fixation and stops driving[97]. Meiotic drivers may bring costly linked genes to fixation, but these costs will not be recoverable via the suppression of the meiotic driver itself, which is uncostly at fixation. Therefore, meiotic drivers will not generate selection for suppression once they have reached fixation[98]. Furthermore, the evolution of meiotic drivers, to increase or decrease their drive strength, will not affect the strength of selection for their suppression, so long as the new mutant drivers reach fixation before a relevant suppressor arises. Finally, because meiotic drivers at fixation do not have a predictable effect on organism phenotype, hybrid crosses, revealing selfish genetic elements, cannot tell apart meiotic drivers that are at fixation from meiotic drivers that are under suppression[99].

The second type of selfish genetic element, which are considered in this study, are "trait distorters". For these SGEs to selfishly propagate themselves, they need to manipulate an organism trait in a specific direction. Our illustrative models focused on a hypothetical trait distorter that manipulated some undefined organism trait, which allowed it to gain a propagation advantage by driving at meiosis. Trait distortion was necessary to facilitate drive, which is what distinguishes our hypothetical trait distorter from a simple meiotic driver. Trait distorters have lasting, predictable effects on the organism after they have reached fixation in the population, compromising individual fitness maximisation. Suppression is therefore favoured even after the trait distorter has reached fixation, and is increasingly favoured as the trait distorter evolves to be more trait-distorting. Finally, hybrid crosses can reveal trait distorters under suppression. If the hybrids express trait distortion whereas the parents do not, the trait distorters were under suppression in the parents[100].

Relation to Eshel (1984) and Eshel (1985)

Eshel[101] highlighted the conflict between individual fitness maximisation and selfish genetic elements. Eshel[98] also pointed out that suppressors of simple meiotic drivers will spread as long as (i) the driver is below fixation, and (ii) the suppressor is unlinked. In doing so, he pointed out that fair meiosis can be stabilised if there is free-recombination between genes. There are a few key differences between our models and the models of meiotic drive suppression developed by Eshel[98] and others[48,102,103].

Eshel[98] modelled a simple meiotic driver and an unlinked suppressor. The driver may exert an individual cost in heterozygous and/or homozygous form. The suppressor completely suppresses drive, at no cost to the individual. However, upon suppression, the individual cost of drive is not recovered. On these assumptions, the suppressor is favoured via individuals that are heterozygous for the driver. For driver-heterozygotes, those individuals that bear the suppressor will have a lower proportion of offspring that inherit the costly driver. As a result, driver-heterozygotes that bear the suppressor will have more grandchildren than driver-heterozygotes that lack the suppressor[48,98]. An implication of this is that, once the driver has gone to fixation and driver-heterozygotes have consequently diminished, there is no further selection on the suppressor. A second implication is that, because the suppressor is cost-free, it will always spread whilst the driver is present and below fixation, regardless of the individual cost or the transmission advantage associated with the driver.

In our illustrative model, and in contrast to Eshel's[98], the individual cost associated with the driver is recovered when the driver is suppressed. As a result of this, the suppressor can be favoured as a direct consequence of recovering the costs associated with the driver. Suppressor selection need not rely on increasing the number of grandchildren of driver-heterozygotes. In our model, suppressors are therefore still favoured even after the driver has gone to fixation. Furthermore, in our model, and in contrast to Eshel's, the act of suppression incurs an individual cost. As

a result of this, suppressors are not universally favoured, but rather, they are only favoured when drivers are sufficiently costly.

Ours and Eshel's[98] model address different biological scenarios. Non-recoverable costs of drive, as assumed by Eshel, are likely to stem from linked deleterious genes, and not from any systematic distortion of individual traits. This scenario applies, for example, to *Segregation Distorter* (*SD*) in *Drosophila melanogaster*, which drives without systematically biasing organism traits. In accordance with Eshel's model, empirical observation of *Segregation Distorter* (*SD*) in natural populations demonstrates that unlinked suppressors spread easily, but only if the driver is below fixation[104].

In contrast, if the individual costs stem directly from the expression of the driver, rather than any unlinked genes, recoverable costs of drive are appropriate[97]. In this case, suppression of the driver also removes the individual level cost. This scenario applies to cases where the meiotic driver is not just a meiotic driver *per se*, but rather, a "trait distorter" that gains the ability to drive at meiosis as a consequence of distorting an organism trait. Our models demonstrate that trait distorters, unlike the simple meiotic drivers considered by Eshel[98], can promote suppressor spread even after they have gone to fixation. As a result, costly trait distorters ($c_{trait}(k)>c_{sup}$) will be suppressed in the evolutionary long term, even if they can reach fixation in the evolutionary short term.

Our models also expand on Eshel[98] in addressing *how likely* it is that a suppressor will spread. Eshel[98] demonstrated that a cost-free unlinked suppressor can spread in response to a costly meiotic driver. Our models account for a cost of suppression, to show that the likelihood that a trait distorter is suppressed correlates with the costliness of the driver to the individual, which serves to limit deviation from individual fitness maximisation.

Supplementary Discussion 3

Relation to Cosmides & Tooby (1981): Coreplicons, Cabal & Commonwealth

An anonymous referee suggested that, were we to extend our models to permit trait distorter introduction at any locus in the genome, rather than at a subsection of loci that are chosen *a priori* (cabal), the resulting trait distortion may be greater. In this section, we explicitly clarify why cabals are defined *a priori* by showing how they follow from the 'coreplicon' concept introduced by Cosmides & Tooby (1981)[6]. We then undertake this suggested modelling extension, showing that the scenario it depicts: (i)) leads to the same results as our models, but (ii) is biologically implausible.

The coreplicon concept

Cosmides & Tooby (1981)[6] pointed out that we can divide a genome up into 'coreplicons'. A coreplicon comprises a collection of loci within the genome that are inherited in the same way, and so share the same maximand. Autosomal loci and X chromosome loci do not form part of the same coreplicon, because the former are transmitted equally through males and females and the latter are transmitted predominantly through females. Coreplicons are assigned, *a priori*, based on inheritance patterns – not on the basis of trait-affecting alleles that have been observed empirically or within the context of a theoretical model. The coreplicon concept has been employed regularly in the study of intragenomic conflict and evolutionary adaptation[52,110,111].

Coreplicons have the potential to be in conflict over organism traits. If, for a given trait, loci within coreplicon *X* are propagated best when the organism trait value is *x*, but loci within coreplicon *Y* are propagated best when the organism trait value is *y*, then the coreplicons have the potential to be in conflict if the current organism trait value is between *x* and *y*. This evolutionary battleground ('potential conflict') is derived *a priori* based on a purely theoretical, first principles optimisation approach, as detailed in Gardner & Úbeda (2017)[53]. The evolutionary battleground for conflict is independent of whether any conflicting, trait-affecting alleles actually exist at any of the loci ('actual conflict')[112].

Sometimes, different coreplicons may form alliances, because they both benefit from a particular kind of trait distortion. For instance, if coreplicon *Z* is propagated best when the organism trait is *z*, where *z* lies in between *x* and *y* but is closer to *x*, coreplicon *Z* may ally with coreplicon *X* if the current organism trait value lies at *y*. Though the coreplicons may ally here, they may disagree over the form of other traits. This is where the concepts of 'cabal' and 'commonwealth' are useful. For example, in humans, cytoplasmic elements are inherited exclusively through females, and X chromosomes are inherited predominantly though not exclusively through females, meaning they represent different coreplicons. However, the coreplicons form a cabal with respect to sex ratio, favouring a female bias.

The cabal / commonwealth concept

The cabal comprises all coreplicons that favour the distortion of a particular trait, along a particular axis, in a particular direction, away from individual fitness maximisation. The commonwealth comprises the remaining coreplicons. Cabals and commonwealths are therefore trait-specific. It is useful, when analysing a specific trait, to partition the genome along these lines, because it is the resolution of this conflict – between the cabal and commonwealth – that gives the evolved deviation of a trait from individual fitness maximisation. Cabals and commonwealths are defined *a priori*, by partitioning and summing up the coreplicons that respectively disfavour and favour the trait distortion under study.

Our models address whether selfish genetic elements can distort organism traits away from individual fitness maximisation, where the 'individual' here really means the majority interest within the parliament of genes[111]. This is why we only considered cabal sizes of up to a half. If the cabal was greater than half of the genome, it would reflect the majority interest within the parliament, so would cease to be a cabal. Our models therefore consider the full range of scenarios depicting potential distortion of organism traits from individual fitness maximisation.

Modelling extension

Having justified our approach, which defines the cabal and commonwealth *a priori*, we now undertake the theoretical exercise suggested by the anonymous reviewer, and allow trait distorters to arise at any locus in the genome, and not just at an *a priori* subsection (cabal).

We first note that this scenario is biologically implausible. In reality, most sites in the genomes of biological organisms *cannot* become trait distorters. Most loci in a genome are unimprinted, vertically inherited and autosomal. Therefore, for an organism approximating individual fitness maximisation, no conceivable distortion of an organism trait could possibly give these loci a propagation advantage. Meiotic drivers or transposons could arise at any of these loci, and the resulting selfish genetic elements could spread through the population as a result. However, *trait distorters* could not arise at these loci – the transmission of alleles at these loci is maximised when the organism trait values are those which lead to individual fitness maximisation[110,111]. The key difference here is between meiotic drive (could be favoured at any locus; selfish benefit does not arise via distorting a trait) and selfish genetic elements that gain a benefit by distorting a trait (such as the specific examples that we consider and model in this paper)[52,53].

Nevertheless, we will imagine a hypothetical organism where any site in its genome could give rise to a trait distorter. The question then becomes: what type of trait distortion is favoured at each locus? It could firstly be the case that each locus gains its selfish propagation advantage by distorting a unique trait, or by distorting a common trait but along a unique dimension (axis) and direction. If this is the case, each locus in the genome would effectively form its own cabal, with a proportional size within the genome approximating zero ($\theta \to 0$). It could alternatively be the case that groups of loci favour the same type of trait distortion (same trait, dimension and direction), meaning proportional cabal sizes can be larger ($\theta > 0$). However, given that the size of any one cabal cannot exceed a half (else that group of loci would cease

to be a cabal), it must logically be the case that (at least two) different types of trait distortion are favoured across the genome.

We now assume that the rate of trait distorter introduction, per generation, per locus, in some genome within the population, is $\rho_{D1.}$ We take the number of loci within a genome to be γ, which means that new trait distorters are introduced into the population every $1/(\rho_{D1}\gamma)$ generations. This is a faster rate than previously considered in our Dynamics models, which was dependent on proportional cabal size ($1/(\theta\rho_{D1}\gamma)$). As was the case in our Dynamics models, the suppressor of a given trait distorter will be expected to arise after a lag of ($1/(1-\theta)\rho_{S1}\gamma$) generations, where ρ_{S1} is the rate of suppressor introduction, per generation, per locus, for any locus situated outside of the target trait distorter's cabal.

So in this new theoretical scenario, compared to our previous Dynamics models, trait distorters are arising at a faster rate, but they are suppressed at the same rate as before. This would apparently suggest that average trait distortion should be more appreciable in this new scenario. However, this is not the case. The rate that trait distorters *that distort a given trait* are introduced is the same as our Dynamics models ($1/(\theta\rho_{D1}\gamma)$). This new formulation appears to favour increased deviation of organisms from individual fitness maximisation, but this is not the case, as the new scenario is implicitly considering the distortion of multiple traits simultaneously.

The distortion of any *given trait* from individual fitness maximisation in this new theoretical scenario is still accurately given by our Dynamics models. Specifically, in

this new theoretical scenario, if trait distorters belonging to the same cabal arise at new loci in the genome very slowly compared to the rate at which gene frequencies equilibrate after trait distorter / suppressor introduction (separation of timescales), the trait that the cabal is attempting to distort assumes an average value, in individuals over evolutionary time, given by Equation 6 in the main text. If trait distorters belonging to the same cabal arise more quickly than this, such that they may co-segregate, the trait that the cabal is attempting to distort assumes an average value that is given by the simulation results of our Dynamics models. This holds regardless of the overall rate of trait distorter introduction across the whole genome.

Therefore, the scenario in which trait distorters may arise at any locus in the genome implicitly refers to a scenario where multiple traits are being distorted and restored simultaneously, in the context of a single model. However, there is no reason why the evolution of distortion and suppression at one trait should be affected by the evolution of distortion and suppression at any other trait. Consequently, the results of the new theoretical scenario converge on our Dynamics models once we consider a single type of trait distortion in isolation. Our models cover the full range of scenarios depicting potential distortion of an organism trait from individual fitness maximisation. The modelling extension, as well as being biologically implausible, provides no additional insight.

Supplementary References

1. Fisher, R. A. *The Genetical Theory of Natural Selection*. (Oxford University Press, 1930).
2. Charnov, E. L. *The Theory of Sex Allocation*. (Princeton University Press, 1982).
3. West, S. *Sex Allocation*. (Princeton University Press, 2009). doi:10.1515/9781400832019
4. Bull, J. J. *Evolution of sex determining mechanisms*. (Benjamin-Cummings Publishing Company, 1983).
5. Jaenike, J. Sex Chromosome Meiotic Drive. *Annual Review of Ecology and Systematics* **32,** 25–49 (2001).
6. Cosmides, L. M. & Tooby, J. Cytoplasmic inheritance and intragenomic conflict. *Journal of Theoretical Biology* **89,** 83–129 (1981).
7. Helleu, Q., Gérard, P. R. & Montchamp-Moreau, C. Sex Chromosome Drive. *Cold Spring Harbor Perspectives in Biology* **7,** a017616–16 (2015).
8. Hickey, W. A. & Craig, G. B. Genetic Distortion of Sex Ratio in a Mosquito Aedes Aegypti. *Genetics* **53,** 1177–& (1966).
9. Policansky, D. & Ellison, J. Sex-Ratio in Drosophila-Pseudoobscura - Spermiogenic Failure. *Science* **169,** 888–+ (1970).
10. Hauschteckjungen, E. & Maurer, B. Sperm Dysfunction in Sex-Ratio Males of Drosophila-Subobscura. *Genetica* **46,** 459–477 (1976).
11. Wu, C. I. Virility Deficiency and the Sex-Ratio Trait in Drosophila-Pseudoobscura. I. Sperm Displacement and Sexual Selection. *Genetics* **105,** 651–662 (1983).

12. Montchamp-Moreau, C. & Joly, D. Abnormal spermiogenesis is associated with the X-linked sex-ratio trait in Drosophila simulans. *Heredity* **79,** 24–30 (1997).

13. Presgraves, D. C., Severance, E. & Wilkinson, G. S. Sex chromosome meiotic drive in stalk-eyed flies. *Genetics* **147,** 1169–1180 (1997).

14. Wu, C. I. Virility Deficiency and the Sex-Ratio Trait in Drosophila-Pseudoobscura. II. Multiple Mating and Overall Virility Selection. *Genetics* **105,** 663–679 (1983).

15. Taylor, D. R., Saur, M. J. & Adams, E. Pollen performance and sex-ratio evolution in a dioecious plant. *Evolution* **53,** 1028–1036 (1999).

16. Wilkinson, G. S. & Sanchez, M. I. Sperm development, age and sex chromosome meiotic drive in the stalk-eyed fly, Cyrtodiopsis whitei. *Heredity* **87,** 17–24 (2001).

17. Wilkinson, G. S., Johns, P. M., Kelleher, E. S., Muscedere, M. L. & Lorsong, A. Fitness effects of X chromosome drive in the stalk-eyed fly, Cyrtodiopsis dalmanni. *Journal of Evolutionary Biology* **19,** 1851–1860 (2006).

18. Angelard, C., Montchamp-Moreau, C. & Joly, D. Female-driven mechanisms, ejaculate size and quality contribute to the lower fertility of sex-ratio distorter males in Drosophila simulans. *BMC Evol Biol* **8,** 326–12 (2008).

19. Price, T. A. R. *et al.* Sex ratio distorter reduces sperm competitive ability in an insect. *Evolution* **62,** 1644–1652 (2008).

20. Thomson, G. J. & Feldman, M. W. Population genetics of modifiers of meiotic drive: IV. On the evolution of sex-ratio distortion. *Theoretical Population Biology* **8,** 202–211 (1975).

21. Hamilton, W. D. Extraordinary sex ratios. A sex-ratio theory for sex linkage and inbreeding has new implications in cytogenetics and entomology. *Science* **156,** 477–488 (1967).

22. Jaenike, J. Sex-Ratio Meiotic Drive in the Drosophila quinaria Group. *The American Naturalist* **148,** 237–254 (1996).

23. Atlan, A., Joly, D., Capillon, C. & Montchamp-Moreau, C. Sex-ratio distorter of Drosophila simulans reduces male productivity and sperm competition ability. *Journal of Evolutionary Biology* **17,** 744–751 (2004).

24. Beckenbach, A. Multiple Mating and the 'Sex-Ratio' Trait in Drosophila pseudoobscura. *Evolution* **35,** 275–281 (1981).

25. Taylor, J. E. & Jaenike, J. Sperm competition and the dynamics of X chromosome drive: stability and extinction. *Genetics* **160,** 1721–1731 (2002).

26. Edwards, A. W. F. The population genetics of 'sex-ratio' in Drosophila pseudoobscura. *Heredity* **16,** 291–304 (1961).

27. Curtsinger, J. W. & Feldman, M. W. Experimental and Theoretical Analysis of the 'Sex-Ratio' Polymorphism in Drosophila Pseudoobscura. *Genetics* **94,** 445–466 (1980).

28. Stalker, H. D. The Genetic Systems Modifying Meiotic Drive in Drosophila Paramelanica. *Genetics* **46,** 177–202 (1961).

29. Klaczko, L. B. & Carvalho, A. B. Autosomal Suppressors of Sex-Ratio in Drosophila-Mediopunctata. *Heredity* **71,** 546–551 (1993).

30. Jaenike, J. Suppression of sex-ratio meiotic drive and the maintenance of Y-chromosome polymorphism in Drosophila. *Evolution* **53,** 164 (1999).

31. Cazemajor, M., Landre, C. & Montchamp-Moreau, C. The Sex-Ratio Trait in

Drosophila simulans: Genetic Analysis of Distortion and Suppression. *Genetics* **147,** 635–642 (1997).

32. Tao, Y., Hartl, D. L. & Laurie, C. C. Sex-ratio segregation distortion associated with reproductive isolation in Drosophila. *Proc Natl Acad Sci USA* **98,** 1–6 (2001).

33. Tao, Y., Masly, J. P., Araripe, L., Ke, Y. & Hartl, D. L. A sex-ratio meiotic drive system in Drosophila simulans. I: an autosomal suppressor. *PLoS Biol* **5,** e292 (2007).

34. Ferree, P. M. & Barbash, D. A. Distorted Sex Ratios: A Window into RNAi-Mediated Silencing. *PLoS Biol* **5,** e303–5 (2007).

35. Hornett, E. A. *et al.* The Evolution of Sex Ratio Distorter Suppression Affects a 25 cM Genomic Region in the Butterfly Hypolimnas bolina. *PLoS Genet* **10,** e1004822–12 (2014).

36. Vaz, S. C. & Carvalho, A. B. Evolution of autosomal suppression of the sex-ratio trait in Drosophila. *Genetics* **166,** 265–277 (2004).

37. Carvalho, Vaz, S. C. & Klaczko, L. B. Polymorphism for Y-linked suppressors of sex-ratio in two natural populations of Drosophila mediopunctata. *Genetics* **146,** 891–902 (1997).

38. Capillon, C. & Atlan, A. Evolution of Driving X Chromosomes and Resistance Factors in Experimental Populations of Drosophila simulans. *Evolution* **53,** 506 (1999).

39. Wu, C. I. The fate of autosomal modifiers of the sex-ratio trait in Drosophila and other sex-linked meiotic drive systems. *Theoretical Population Biology* **24,** 107–120 (1983).

40. Atlan, A., Capillon, C., Derome, N., Couvet, D. & Montchamp-Moreau, C. The Evolution of Autosomal Suppressors of Sex-Ratio Drive in Drosophila Simulans. *Genetica* **117,** 47–58 (2003).

41. Rood, E. S. & Freedberg, S. Intragenomic conflict produces sex ratio dynamics that favor maternal sex ratio distorters. *Ecol Evol* **6,** 8085–8093 (2016).

42. Bastide, H., Gérard, P. R., Ogereau, D., Cazemajor, M. & Montchamp-Moreau, C. Local dynamics of a fast-evolving sex-ratiosystem in Drosophila simulans. *Molecular Ecology* **22,** 5352–5367 (2013).

43. Werren, J. H. The coevolution of autosomal and cytoplasmic sex ratio factors. *Journal of Theoretical Biology* **124,** 317–334 (1987).

44. Maynard Smith, J. & Price, G. R. The Logic of Animal Conflict. *Nature* **246,** 15–18 (1973).

45. Tao, Y. *et al.* A sex-ratio meiotic drive system in Drosophila simulans. II: an X-linked distorter. *PLoS Biol* **5,** e293 (2007).

46. Wu, C. I. & Beckenbach, A. T. Evidence for Extensive Genetic Differentiation Between the Sex-Ratio and the Standard Arrangement of Drosophila-Pseudoobscura and Drosophila-Persimilis and Identification of Hybrid Sterility Factors. *Genetics* **105,** 71–86 (1983).

47. Dyer, K. A., Charlesworth, B. & Jaenike, J. Chromosome-wide linkage disequilibrium as a consequence of meiotic drive. *Proc Natl Acad Sci USA* **104,** 1587–1592 (2007).

48. Crow, J. F. Why is mendelian segregation so exact? *Bioessays* **13,** 305–312 (1991).

49. Wild, G. & Traulsen, A. The different limits of weak selection and the evolutionary dynamics of finite populations. *Journal of Theoretical Biology* **247,** 382–390 (2007).

50. Peters, J. The role of genomic imprinting in biology and disease: an expanding view. *Nature Publishing Group* **15,** 517–530 (2014).

51. Hamilton, W. D. The genetical evolution of social behaviour. I. *Journal of Theoretical Biology* **7,** 1–16 (1964).

52. Burt, A. & Trivers, R. *Genes in Conflict*. (Harvard University Press, 2006). doi:10.4159/9780674029118

53. Gardner, A. & Úbeda, F. The meaning of intragenomic conflict. *Nature Ecology & Evolution* **1,** 1–9 (2017).

54. Haig, D. *Genomic Imprinting and Kinship*. (Rutgers University Press, 2002).

55. Úbeda, F. & Gardner, A. A model for genomic imprinting in the social brain: juveniles. *Evolution* **64,** 2587–2600 (2010).

56. Queller, D. C. Theory of genomic imprinting conflict in social insects. *BMC Evol Biol* **3,** 15 (2003).

57. Haig, D. Parental antagonism, relatedness asymmetries, and genomic imprinting. *Proceedings of the Royal Society B: Biological Sciences* **264,** 1–6 (1997).

58. Haig, D. Genomic imprinting, sex-biased dispersal, and social behavior. *Ann. N. Y. Acad. Sci.* **907,** 149–163 (2000).

59. Úbeda, F. & Gardner, A. A model for genomic imprinting in the social brain: adults. *Evolution* **65,** 462–475 (2011).

60. Úbeda, F. & Gardner, A. A model for genomic imprinting in the social brain:

elders. *Evolution* **66,** 1567–1581 (2012).

61. Haig, D. Coadaptation and conflict, misconception and muddle, in the evolution of genomic imprinting. *Heredity* **113,** 96–103 (2013).

62. Kondoh, M. & Higashi, M. Reproductive Isolation Mechanism Resulting from Resolution of Intragenomic Conflict. *The American Naturalist* **156,** 511–518 (2000).

63. Wilkins, J. F. & Haig, D. Genomic imprinting of two antagonistic loci. *Proceedings of the Royal Society B: Biological Sciences* **268,** 1861–1867 (2001).

64. Wilkins, J. F. Genomic imprinting and conflict-induced decanalization. *Evolution* **65,** 537–553 (2011).

65. Wilkins, J. F. Antagonistic coevolution of two imprinted loci with pleiotropic effects. *Evolution* **64,** 142–151 (2010).

66. Gregg, C. *et al.* High-Resolution Analysis of Parent-of-Origin Allelic Expression in the Mouse Brain. *Science* **329,** 643–648 (2010).

67. Galbraith, D. A. *et al.* Testing the kinship theory of intragenomic conflict in honey bees (Apis mellifera). *Proc Natl Acad Sci USA* **113,** 1020–1025 (2016).

68. Foster, K. R. Diminishing returns in social evolution: the not-so-tragic commons. *Journal of Evolutionary Biology* **17,** 1058–1072 (2004).

69. Haig, D. The Kinship Theory of Genomic Imprinting. *Annual Review of Ecology and Systematics* **31,** 9–32 (2000).

70. Jacob, S., McClintock, M. K., Zelano, B. & Ober, C. Paternally inherited HLA alleles are associated with women's choice of male odor. *Nat Genet* **30,** 175–

179 (2002).

71. Isles, A. R. *et al.* A possible role for imprinted genes in inbreeding avoidance and dispersal from the natal area in mice. *Proceedings of the Royal Society B: Biological Sciences* **269,** 665–670 (2002).

72. Isles, A. R., Baum, M. J., Ma, D., Keverne, E. B. & Allen, N. D. Urinary odour preferences in mice. *Nature* **409,** 783–784 (2001).

73. Hager, R. & Johnstone, R. A. The genetic basis of family conflict resolution in mice. *Nature* **421,** 533–535 (2003).

74. Gardner, A., West, S. A. & Wild, G. The genetical theory of kin selection. *Journal of Evolutionary Biology* **24,** 1020–1043 (2011).

75. Rousset, F. *Genetic Structure and Selection in Subdivided Populations (MPB-40).* (Princeton University Press, 2013).

76. Gardner, A. & Ross, L. Mating ecology explains patterns of genome elimination. *Ecol Lett* **17,** 1602–1612 (2014).

77. Burt, A. & Trivers, R. Genetic conflicts in genomic imprinting. *Proceedings of the Royal Society B: Biological Sciences* **265,** 2393–2397 (1998).

78. Kafri, T., Gao, X. H. & Razin, A. Mechanistic Aspects of Genome-Wide Demethylation in the Preimplantation Mouse Embryo. *Proc Natl Acad Sci USA* **90,** 10558–10562 (1993).

79. West, S. A., Diggle, S. P., Buckling, A., Gardner, A. & Griffin, A. S. The Social Lives of Microbes. *Annu. Rev. Ecol. Evol. Syst.* **38,** 53–77 (2007).

80. Frank, S. A. A general model of the public goods dilemma. *Journal of Evolutionary Biology* **23,** 1245–1250 (2010).

81. West, S. A. & Buckling, A. Cooperation, virulence and siderophore production

in bacterial parasites. *Proceedings of the Royal Society B: Biological Sciences* **270,** 37–44 (2003).

82. Mc Ginty, S. E., Lehmann, L., Brown, S. P. & Rankin, D. J. The interplay between relatedness and horizontal gene transfer drives the evolution of plasmid-carried public goods. *Proceedings of the Royal Society B: Biological Sciences* **280,** 20130400–20130400 (2013).

83. Mc Ginty, S. É., Rankin, D. J. & Brown, S. P. Horizontal gene transfer and the evolution of bacterial cooperation. *Evolution* **65,** 21–32 (2011).

84. Dimitriu, T. *et al.* Genetic information transfer promotes cooperation in bacteria. *Proc Natl Acad Sci USA* **111,** 11103–11108 (2014).

85. Dimitriu, T. *et al.* Indirect Fitness Benefits Enable the Spread of Host Genes Promoting Costly Transfer of Beneficial Plasmids. *PLoS Biol* **14,** e1002478–28 (2016).

86. Dimitriu, T. *et al.* Selection of horizontal gene transfer through public good production. *bioRxiv* **106,** 1

87. Niehus, R., Mitri, S., Fletcher, A. G. & Foster, K. R. Migration and horizontal gene transfer divide microbial genomes into multiple niches. *Nat. Comun.* **6,** 1–9 (2015).

88. Ghaly, T. M. & Gillings, M. R. Mobile DNAs as Ecologically and Evolutionarily Independent Units of Life. *Trends in Microbiology* **26,** 904–912 (2018).

89. Smith, J. The social evolution of bacterial pathogenesis. *Proceedings of the Royal Society B: Biological Sciences* **268,** 61–69 (2001).

90. Rankin, D. J., Rocha, E. P. C. & Brown, S. P. What traits are carried on mobile genetic elements, and why? *Heredity* **106,** 1–10 (2011).

91. Nogueira, T. *et al.* Horizontal Gene Transfer of the Secretome Drives the Evolution of Bacterial Cooperation and Virulence. *Current Biology* **19,** 1683–1691 (2009).

92. Johnson, L. J. The Genome Strikes Back: The Evolutionary Importance of Defence Against Mobile Elements. *Evolutionary Biology* **34,** 121–129 (2007).

93. Mc Ginty, S. É. & Rankin, D. J. The evolution of conflict resolution between plasmids and their bacterial hosts. *Evolution* **66,** 1662–1670 (2012).

94. Ghoul, M. *et al.* Pyoverdin cheats fail to invade bacterial populations in stationary phase. *Journal of Evolutionary Biology* **29,** 1–9 (2016).

95. Gardner, A. & Welch, J. J. A formal theory of the selfish gene. *Journal of Evolutionary Biology* **24,** 1801–1813 (2011).

96. Grafen, A. The formal darwinism project in outline. *Biol Philos* **29,** 155–174 (2014).

97. Zanders, S. E. & Unckless, R. L. Fertility Costs of Meiotic Drivers. *Current Biology* **29,** R512–R520 (2019).

98. Eshel, I. Evolutionary Genetic Stability of Mendelian Segregation and the Role of Free Recombination in the Chromosomal System. *The American Naturalist* **125,** 412–420 (1985).

99. Corbett-Detig, R., Medina, P., Frérot, H., Blassiau, C. & Castric, V. Bulk pollen sequencing reveals rapid evolution of segregation distortion in the male germline of Arabidopsis hybrids. *Evolution Letters* **129,** 1393–11 (2019).

100. Blows, M. W., Berrigan, D. & Gilchrist, G. W. Rapid evolution towards equal sex ratios in a system with heterogamety. *Evol Ecol Res* 277–283 (1999).

101. Eshel, I. Are intragametic conflicts common in nature? Do they represent an

important factor in evolution? *Journal of Theoretical Biology* **108,** 159–162 (1984).

102. Feldman, M. W. & Otto, S. P. A Comparative Approach to the Population-Genetics Theory of Segregation Distortion. *The American Naturalist* **137,** 443–456 (1991).

103. Prout, T., Bundgaard, J. & Bryant, S. Population genetics of modifiers of meiotic drive I. The solution of a special case and some general implications. *Theoretical Population Biology* **4,** 446–465 (1973).

104. Larracuente, A. M. & Presgraves, D. C. The selfish Segregation Distorter gene complex of Drosophila melanogaster. *Genetics* **192,** 33–53 (2012).

105. Bernardo Carvalho, A., Koerich, L. B. & Clark, A. G. Origin and evolution of Y chromosomes: Drosophila tales. *Trends in Genetics* **25,** 270–277 (2009).

106. Matthews, B. B. *et al.* Gene Model Annotations for Drosophila melanogaster: Impact of High-Throughput Data. *G3 (Bethesda)* **5,** 1721–1736 (2015).

107. Taanman, J. W. The mitochondrial genome: structure, transcription, translation and replication. *Biochim. Biophys. Acta* **1410,** 103–123 (1999).

108. Zerbino, D. R. *et al.* Ensembl 2018. *Nucleic Acids Research* **46,** D754–D761 (2017).

109. Pertea, M. *et al.* CHESS: a new human gene catalog curated from thousands of large-scale RNA sequencing experiments reveals extensive transcriptional noise. *Genome Biol.* **19,** 1–14 (2018).

110. Haig, D. Genetic dissent and individual compromise. *Biol Philos* **29,** 233–239 (2014).

111. Grafen, A. Optimization of inclusive fitness. *Journal of Theoretical Biology*

238, 541–563 (2006).

112. Queller, D. C. & Strassmann, J. E. Evolutionary Conflict. *Annu. Rev. Ecol. Evol. Syst.* **49,** annurev–ecolsys–110617–062527–21 (2018).
